# Population structure of Nepali spring wheat (*Triticum aestivum* L.) germplasm

**DOI:** 10.1186/s12870-020-02722-8

**Published:** 2020-11-23

**Authors:** Kamal Khadka, Davoud Torkamaneh, Mina Kaviani, Francois Belzile, Manish N. Raizada, Alireza Navabi

**Affiliations:** 1 Department of Plant Agriculture, University of Guelph, Guelph, Ontario N1G 2W1 Canada; 2 Département de Phytologie, Université Laval, Québec City, QC G1V 0A6 Canada; 3 Institut de Biologie Intégrative et des Systèmes (IBIS), Université Laval, Québec City, QC Canada

**Keywords:** Nepal, Landraces, Genotype-by-sequencing (GBS), Genetic diversity, Population structure, Linkage disequilibrium

## Abstract

**Background:**

Appropriate information about genetic diversity and population structure of germplasm improves the efficiency of plant breeding. The low productivity of Nepali bread wheat (*Triticum aestivum* L.) is a major concern particularly since Nepal is ranked the 4th most vulnerable nation globally to climate change. The genetic diversity and population structure of Nepali spring wheat have not been reported. This study aims to improve the exploitation of more diverse and under-utilized genetic resources to contribute to current and future breeding efforts for global food security.

**Results:**

We used genotyping-by-sequencing (GBS) to characterize a panel of 318 spring wheat accessions from Nepal including 166 landraces, 115 CIMMYT advanced lines, and 34 Nepali released varieties. We identified 95 K high-quality SNPs. The greatest genetic diversity was observed among the landraces, followed by CIMMYT lines, and released varieties. Though we expected only 3 groupings corresponding to these 3 seed origins, the population structure revealed two large, distinct subpopulations along with two smaller and scattered subpopulations in between, with significant admixture. This result was confirmed by principal component analysis (PCA) and UPGMA distance-based clustering. The pattern of LD decay differed between subpopulations, ranging from 60 to 150 Kb. We discuss the possibility that germplasm explorations during the 1970s–1990s may have mistakenly collected exotic germplasm instead of local landraces and/or collected materials that had already cross-hybridized since exotic germplasm was introduced starting in the 1950s.

**Conclusion:**

We suggest that only a subset of wheat “landraces” in Nepal are authentic which this study has identified. Targeting these authentic landraces may accelerate local breeding programs to improve the food security of this climate-vulnerable nation. Overall, this study provides a novel understanding of the genetic diversity of wheat in Nepal and this may contribute to global wheat breeding initiatives.

**Supplementary Information:**

**Supplementary information** accompanies this paper at 10.1186/s12870-020-02722-8.

## Key message

The study explores the population structure and genetic diversity of spring wheat germplasm in Nepal, not reported previously. The findings were unexpected and provide a novel understanding of the germplasm.

## Background

A significant challenge faced by modern plant breeders is the need to improve crop yield in the wake of the ever-increasing human population while combating the consequences posed by climate change on crop productivity [1]. The current world population of ~ 8 billion in 2019 is expected to exceed 9.6 billion in 2050 which means that food production must increase by at least 33% to meet the growing demand [2]. However, to eradicate global hunger by 2030 as targeted by the Sustainable Development Goals of the United Nations, the current state of research and development does not seem to be able to meet this important challenge [3]. While there is limited scope for increasing the area under food production, yield increase through genetic improvement is the key means to overcome issues related to future food crises. Current studies related to climate change outline the increasing occurrence of heat, cold and drought stresses that are detrimental to agricultural crops [4]. This situation is posing numerous challenges for improving the production of crops including bread wheat (*Triticum aestivum* L.). Bread wheat is the third most important staple cereal crop in the world with a global production of 757 million metric tonnes in 2017 [5]. Globally, wheat provides 41% of the total cereal calorie intake, constituting 35% of the cereal calorie intake in developing countries, and 74% in developed countries [6]. Overall, wheat ranks second globally in terms of dietary intake, and a large majority of the crop (68%) is used as food while approximately 19% is for feed and biofuels [7].

Similar to the global context, wheat is also one of the major cereals in Nepal. The area under wheat cultivation increased > 5-fold in Nepal since 1960 [8] and today constitutes 1/5th of the nation’s cereal acreage [9]. The current yield of wheat in Nepal is 2.2 t/ha [9], compared to 3.1 t/ha in the United States [10] and 3.2 t/ha in nearby countries such as India [10]. The demand for wheat in Nepal is expected to grow by ~ 890 thousand metric tonnes by 2030 [11]. However, Nepal is ranked as the 4th most vulnerable nation globally to climate change, making it especially vulnerable to drought and other climate-related hazards [12]. This scenario suggests that Nepal can benefit from further advances in wheat breeding. To accelerate such efforts, a thorough understanding of genetic diversity and population structure of available wheat germplasm can potentially aid in the more efficient deployment of available genetic resources [13]. Germplasm molecular characterization can point to unique sources of alleles in the population and prevent genetically redundant germplasm from being used as parents in breeding programs [14]. Furthermore, such analysis reveals past geographic flows of germplasm, their degree of genetic isolation and/or mixing [15, 16]. In addition, such analysis can uncover potential mistakes in germplasm passport information. In this context, the analysis of the genetic diversity of the existing wheat population in Nepal can be valuable. The population structure of Nepali spring wheat has not previously been reported.

The evolution of modern bread wheat through the hybridization of donor species with narrow genetic variation created a genetic bottleneck leading to narrow genetic diversity [17]. Narrow genetic variation is also the result of bottlenecks during the domestication process combined with intensive breeding efforts in the past few decades [18, 19]. Therefore, wheat breeders are always interested in opportunities to diversify and widen the genetic diversity of the crop. Landraces which have been grown by traditional farmers mostly under low input conditions are regarded as one of the major sources of germplasm diversity [20]. Locally adapted elite germplasm, the result of modern breeding programs, can be used for targeted introduction of specific alleles [18–20]. In Nepal, hundreds of landraces are available in the National Agricultural Genetic Resources Centre (NAGRC) which is the national genebank for Nepal, belonging to the Nepal Agriculture Research Council (NARC). Since the 1950s, Nepal has been introducing elite germplasm from The International Maize and Wheat Improvement Center (CIMMYT, Mexico), which have formed the foundation of varieties released by The National Wheat Research Program (NWRP) of Nepal.

Genetic diversity characterization at the molecular level benefits from a genome sequence. Bread wheat has a large genome (~ 17,000 Mb) [21] of which ~ 80% is comprised of repetitive sequences [22, 23]. The annotated reference genome sequence of hexaploid bread wheat was released in 2018 [24], covering all 21 chromosomes and included 107,891 high-confidence genes. The presence of a high-quality reference genome has opened avenues to exploit available wheat genetic resources using modern tools and approaches. The genome sequence has enabled the development of high-density genome-wide markers [25]. Genotyping-by-sequencing (GBS) is one such next-generation sequencing (NGS) based technique that is capable of producing high-density genome-wide markers [26, 27]. Among the various methods used to achieve complexity reduction, the GBS method is more efficient considering the cost, ease of handling, and lower number of purification steps [28, 29]. It reduces the genome complexity significantly and creates more homogenous libraries for sequencing. Since allopolyploidy and large genome size are the two key factors that have deterred the development of molecular markers in wheat, the use of GBS markers presents a step forward to achieve current and future genomic exploration. GBS markers include SNP markers which are widely used in genetic studies requiring large sets of markers such as the determination of population structure, QTL mapping, marker-trait association, genomic selection and map-based cloning [30, 31].

This study aims to contribute to Nepali as well as global plant breeding efforts by appraising the genetic diversity and population structure of Nepali spring wheat. Here, we used GBS derived SNPs to evaluate a panel of 318 spring wheat accessions including landraces, released varieties, and advanced breeding lines. The major objectives of the study were: (i) to characterize the panel for genetic diversity and linkage disequilibrium (LD), and (ii) to identify the underlying basis of the population structure of the Nepali wheat genetic materials.

## Results

### The density of polymorphic GBS markers differs among the genomes of bread wheat

**Fig. 1:**
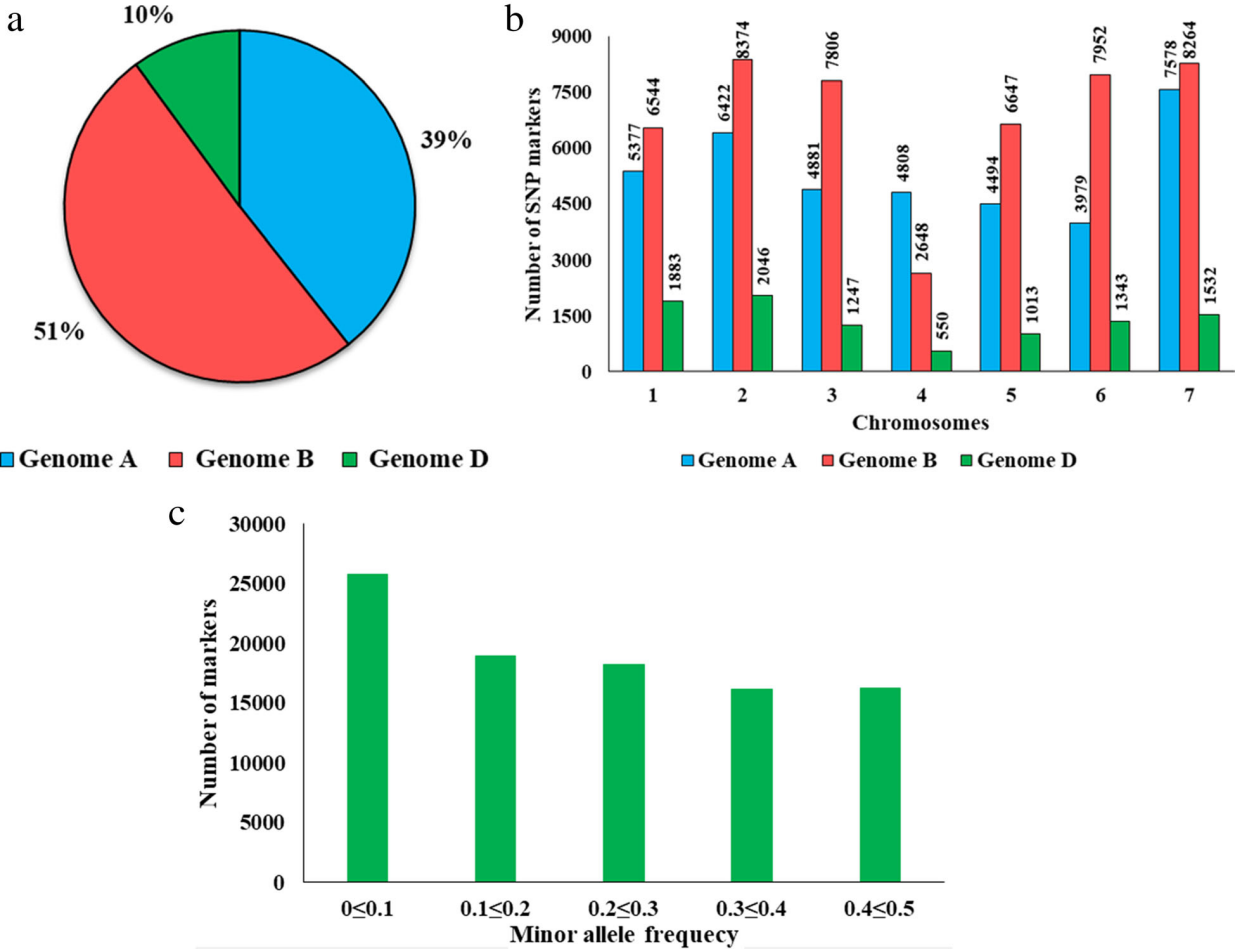
Analysis of SNP markers in the Nepali Wheat Diversity Panel (NWDP). (**a**) Proportionate distribution of SNP markers across genomes A, B, and D in 318 spring wheat genotypes. (**b**) Distribution of 95,388 single nucleotide polymorphism (SNP) markers in the three wheat sub-genomes (A, B and D) across all 21 chromosomes from the 318 spring wheat genotypes. (**c**) Distribution of minor allele frequency for 95,388 SNP markers in the 318 spring wheat genotypes

We performed genotyping-by-sequencing (GBS) of the 318 accessions (Additional file 1: Table S1) and obtained ~ 800 million reads, for an average of 2.5 million reads per accession. Raw reads were processed with the Fast-GBS pipeline to call SNPs. After the imputation of missing data, we obtained a final dataset of 95,388 polymorphic markers across the A, B and D genomes (Additional file 2: Table S2, Fig. 1a, b). The highest proportion of SNP markers (51%) was derived from the B genome followed by the A genome (39%) and the remaining 10% from the D genome. The number of SNP markers per chromosome ranged from 550 (4D) to 8374 (2B) (Additional file 2: Table S2, Fig. 1b). In each of the genomes, the lowest and highest number of markers identified per genome, respectively, ranged from 3979 (6A) to 7578 (7A), 2648 (4B) to 8374 (2B) and 550 (4D) to 2046 (2D), respectively (Additional file 2: Table S2, Fig. 1b). Around 26% of the SNPs (25,820) in this catalog had a minor allele that could be called rare (MAF < 0.1) (Fig. 1c).

### The populations from different sources vary in genetic diversity

The genetic diversity within the NWDP was assessed using two different indices: nucleotide diversity and Tajima’s D. The nucleotide diversity of the panel was measured in each of the three components of the NWDP (the three Canadian genotypes were included in the group “released varieties”). As expected, the highest genetic diversity was observed among the landraces (166) followed by CIMMYT lines (115) and released varieties (37) (Table 1).

**Table 1:** Genetic diversity within the three components of the Nepali Wheat Diversity Panel

Population (#accessions)	SNPs per accession	SNP count	Pi	Tajima’s D
Landraces (166)	571	94,794	6.11E-04	2.14E-03
CYMMYT lines (115)	798	91,794	5.60E-04	1.90E-03
Released varieties (37)^a^	2184	80,822	5.34E-04	1.41E-03

^a^*Note: It includes 3 Canadian varieties*

### The subgroup separation did not correlate to the seed source

**Fig. 2:**
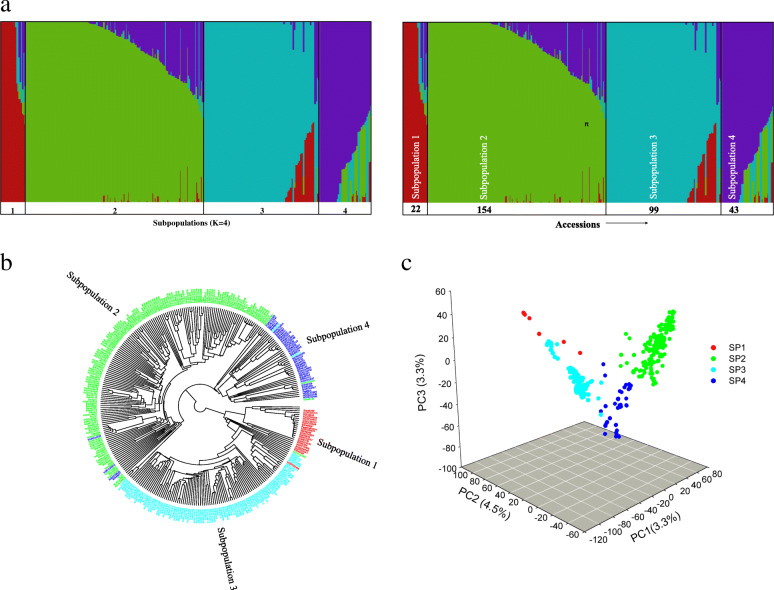
Population structure analysis of the NWDP. **a** Estimated population structure of 318 spring wheat genotypes from Nepal on K = 4. Columns represent individual wheat accessions, while the length represents the proportion of each subpopulation (indicated by the colour) belonging to that accession. **b** Dendrogram based on cluster analysis using pairwise genetic distances. **c** Principal component analysis (PCA) using 95 K GBS markers. The labels SP1, SP1, SP3 and SP4 correspond to the subpopulations 1, 2, 3 and 4

The fastSTRUCTURE analysis determined that four subpopulations (K = 4) was the optimal number of clusters for the 318 accessions in the NWDP using 95 K high-quality SNP markers (Fig. 2a). Among these four subpopulations, subpopulations 2 and 3 were distinct and large while the other two (1 and 4) were found to be smaller. Results revealed that subpopulations 1, 2, 3 and 4 include 22, 154, 99 and 43 accessions, respectively. However, neither the number of sub-populations nor the assignment of accessions perfectly reflected the existence and composition of the three types of accessions included in the NWDP (Fig. 2a, Additional file 1: Table S1).

Clustering analysis was then performed using UPGMA and based on the estimates of relatedness or kinship. The resulting dendrogram (Fig. 2b) was obtained using the estimated genetic distance that provided the placement of each accession in a certain cluster and order. The clusters were in high agreement with the population structure described above (Fig. 2a), although there were some instances where an accession assigned to a subgroup was not grouped with other individuals of the same subgroup (indicated by a colour label different than the respective subpopulation in Fig. 2a, Fig. 2b; Additional file 3: Table S3).

The PCA conducted to validate the results obtained above confirmed the existence of two large and distinct subpopulations as well as two smaller and more overlapping subpopulations (Fig. 2c). Three PCs were used for this analysis, where the three PCs accounted for approximately 14% of the variation. The result showed that the biplots of PC1, PC2, and PC3 separated the genotypes into two distinct groups (subpopulations 2 and 3) while the other two groups (subpopulations 1 and 4) were observed scattered somewhere in between. The accessions in subpopulation 3 appeared more distinct whereas 2 and 4 appeared slightly closer to one another despite subpopulation 2 being distinct. The accessions appeared tightly clustered within subpopulations 2 and 3, while subpopulations 1 and 4 were smaller and more scattered. The smallest cluster, i.e. subpopulation 1, appeared to be highly scattered compared to the other subpopulations (Fig. 2c).

### Linkage disequilibrium decayed more rapidly in the whole population

**Fig. 3:**
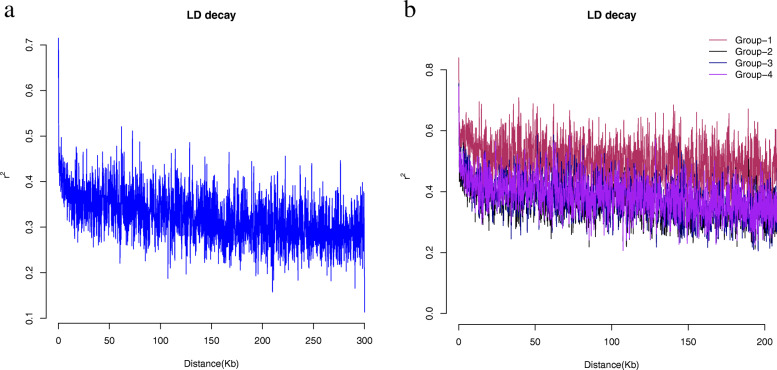
Linkage disequilibrium (LD) analysis of the NWDP. **a** The LD decay (*r*^*2*^) over physical distance for the whole population. **b** The LD decay (*r*^*2*^) over physical distance for the subpopulations where: Group-1 = subpopulation 1, Group-2 = subpopulation 2, Group-3 = subpopulation 3 and Group-4 = subpopulation 4

The analysis of LD decay was performed for the whole panel and separately for each subpopulation derived from the population structure analysis. The estimated allele frequency correlations (*r*^*2*^) were plotted against the physical distance of the loci pairs to assess the pattern of LD decay. On average, the LD (*r*^*2*^ = 0.72) had decayed by half (*r*^*2*^ = 0.36) at a physical distance of ~ 60 Kb for the whole population (Fig. 3a).

The result also showed variable LD patterns for the four different subpopulations (Fig. 3b). The LD observed was highest for subpopulation 1 (*r*^*2*^ = 0.85) followed by subpopulation 3 (*r*^*2*^ = 0.75), subpopulation 4 (r^2^ = 0.75) and then subpopulation 2 (r^2^ = 0.6). For subpopulation 1, the LD declined to half at a physical distance greater than 150 Kb. Similarly, for subpopulation 2, the LD declined to half of its value (*r*^*2*^ = 0.3) at a physical distance of around ~ 75 Kb. In subpopulation 3, the LD decayed to half at a physical distance of ~ 90 Kb. The pattern of LD decay in subpopulation 4 was similar to that of subpopulation 3, i.e. ~ 90 Kb. Thus, the results evidently exhibit differences in *r*^*2*^ values among the whole population and the identified subpopulations. On average, subpopulations 1 and 4 had higher *r*^*2*^ values while the whole population and subpopulation 2 had the lower *r*^*2*^ values. Similarly, LD decay of the whole population was lower (~ 60 Kb) compared to all the four subpopulations.

## Discussion

### The population structure may be governed by the extensive introduction of germplasm into Nepal

The wheat accessions used in this study are representative of spring wheat diversity in Nepal. The analysis of population structure assigned the 318 wheat accessions of the Nepal Wheat Diversity Panel (NWDP) into four subpopulations (two large and two small subpopulations scattered in between). All the three methods used (population structure, UPGMA clustering, and PCA) consistently led to this grouping. The consistency of grouping using these methods has also been observed in earlier studies [32–34]. The differentiation of the population into different subpopulations by fastSTRUCTURE is based on frequencies of relatedness of the genotypes to each of the subpopulations as hypothesized [14, 35, 36]. Similarly, the UPGMA clustering separates the population into different subpopulations based on genetic distances [37] while PCA illustrates the subpopulation differentiation based on genetic distances [38].

A priori, we expected 3 groupings, corresponding to the seed origin, with the first one comprising the landraces from Nepal, and the others including CIMMYT lines and commercially released Nepali varieties. This expectation was based on the conviction that the landraces grown in diverse areas of Nepal, a mountainous nation with diverse ethnic groups, are genetically more diverse than newly introduced modern cultivars [20, 39]. However, contrary to our expectation, the result obtained did not yield such distinct clusters based on the sources of the accessions, but rather admixture [40] was observed among all four subpopulations. Substantial admixture in the population was indicated by the first principal component explaining only 6.3% of the total genotypic variation. We observed that subpopulation 2 had the highest proportion of CIMMYT lines (60%) and released varieties (65%), whereas subpopulation 3 had the highest proportion of landraces (42%) (Additional file 4: Table S4). Similarly, observations from the Q-matrix, an output of Bayesian clustering in fastSTRUCTURE, showed that there are many accessions in each of the four subgroups which are not related (frequency of relatedness > 99%) to other individuals from other groups (Additional file 3: Table S3). Specifically, among 99 accessions grouped into subpopulation 3, 69 accessions are not related to the individuals from other groups, and 49 of these 69 accessions are the landraces. The result suggests that these 49 accessions could be the authentic landraces (Additional file: Table S1; Additional file 3: Table S3). However, in subpopulation 2, among 154 accessions, 56 are not related to accessions from other subpopulations, and 28 of them are CIMMYT lines followed by landraces and the released Nepali varieties. Interestingly, the result showed that some accessions with the same, or at least one or more, common ancestor(s), are clustered in the same group. For example, CIMMYT lines BW49342, BW49351, BW49392, BW49394 and BW49954 have a common ancestor “KACHU” and they are grouped in subpopulation 2 (Additional file: Table S1). Similarly, the released variety Tilottama, and the CIMMYT lines BW48137, BW49108 and BW49456, all of which share the common ancestor “VIVITSI”, are grouped in subpopulation 2. With regards to the Canadian accessions, none of the three accessions had any common ancestors with other accessions in the NWDP. The Canadian accession “Norwell” was grouped in subpopulation 2 and it could possibly be that its ancestors had some association with other CIMMYT germplasm not included in this study (Additional file: Table S1).

The unexpected population structure (4 subpopulations not 3; admixture) results may be due to the numerous factors that influence the structure of a germplasm population such as age of the variety, activities of plant breeders [41, 42], geographical origin [36, 38, 43, 44], market class [37, 45] and ploidy level [39]. Wheat has been growing in Nepal (mostly in the western hills) from ancient times [46]. But formal wheat breeding by the public sector started in Nepal in 1951; Lerma 52 was the first wheat variety released in Nepal for commercial cultivation in 1960 [46]. Until 1960, wheat was a minor crop in Nepal; mostly the landraces and some Indian varieties were predominant in about 100,000 ha area under wheat cultivation [46, 47]. But the scenario changed dramatically in the following decades as a large number of semi-dwarf modern varieties were introduced into Nepal from the beginning of the mid-1960s simultaneous to the government promoting the cultivation of the improved varieties [48]. Wheat germplasm was sourced in Nepal mainly from CIMMYT, Mexico and India [45, 46, 48] and USAID [45]. The wheat area expanded by over 500% between 1960 and 1990 [8] which is also known as the “Green Revolution” [48]. It is also stated that some of the popularly grown wheat varieties before the mid-1960s were also the modern varieties developed by Indian and Mexican wheat breeding programs [8]. Thus, from a minor crop in the 1950s [48], wheat has now become one of the major cereals in Nepal, contributing to 22% of the national cereal coverage [9]. This situation likely resulted in a heavy decline in the cultivation of Nepali landraces.

Examining the 43 varieties released in the country since 1960, Nepal has been highly dependent on foreign germplasm for variety development [46]. On average, the current adoption of improved varieties in Nepal is approximately 97% [46, 49]. Thirty-five wheat varieties released in Nepal until 2001 used a total of 89 ancestors from 22 countries while these did not include any Nepali germplasm [45]. Among the released wheat varieties in Nepal until 2016, approximately 80% of them do not have a Nepali origin (Additional file: Table S1) while the ancestors of these released varieties are notably from the United States (13%), India (13%) and France (12%), Argentina (6%) and Italy (6%) [50]. This evidence clearly highlights the dominance of foreign materials in the wheat germplasm pool of Nepal.

The NAGRC was established in 2010 and it has been working on maintaining agricultural genetic resources through characterization, evaluation, and identification of valuable traits. There are about 1700 wheat accessions collected and maintained by NAGRC [51]. Altogether 18 germplasm collection programs were carried out to collect different crop species including wheat, while only two explorations were conducted to collect wheat genetic resources from Western Nepal [45] which is potentially the major source of diverse Nepali landraces. These genetic resources were collected from different altitudes ranging from 720 to 3353 m above sea level. A large portion of these collections was not from standing crops but from the farmers’ granaries due to difficulties associated with the rugged terrain and inaccessible remote agro-ecological conditions of Nepal [51]. Therefore, the collection process, by chance, could have resulted in the duplication of genetic resources, and furthermore, some of the passport information collected during the germplasm exploration could be misleading, i.e. some of the collections may not have been the “authentic” landraces. Here, the argument is that there is a high probability that germplasm explorations during the 1970s, 1980s, and 1990s could have mistakenly collected and labelled exotic germplasm as local landraces because the farmers had been growing them for decades (after the introduction of exotic materials in 1950s and early 1960s). There is a second potential cause: though Nepal is not one of the centres of origin for wheat, the germplasm spread and evolved collinearly with human migration [52]. In this context, one of the sources of exotic wheat genetic diversity in Nepal could be associated with seasonal migration of farmers from far-western and mid-western Nepal back and forth to/from North-western India, which continues for at least 2–3 generations for many families [53]. Historical farmer-led sharing of seeds between India and Nepal, combined with formal introduction of CIMMYT materials, may be multiplying the distortions within the Nepali wheat population structure, because there has been extensive collaboration and use of Indian wheat genetic resources in international wheat breeding programs (e.g. CIMMYT), enabled by the reasonable assumption that traits adapted to northern Indian would also be beneficial to Nepal. The latter statement is evidenced by the observation that out of 35 wheat varieties released in Nepal until 2001, 16 of them were shown to have an Indian origin, while 14 were from Mexico, 4 from Nepal and the remaining 1 from Kenya (Additional file: Table S1) [45]. Nepal shares a border with China, and an interesting question is the extent to which Chinese wheat genetic resources have influenced Nepalese wheat. The influence of Chinese wheat on the Nepali wheat germplasm pool has not been reported extensively. Nevertheless, based on the information available, at least one of the released varieties from Nepal (Kanti, released in 1997) has one of its ancestors (FUFAN17) as originating from China [46]. Whereas the southern border with India is freely open and accessible, the Himalayas interrupts the flow of people and seeds between China and Nepal, and moreover the limited crossing points between the two nations are more tightly controlled. In terms of the extensive admixture between the “landraces” and exotic germplasm, since the germplasm collections occurred from the 1970s–1990s, whereas CIMMYT germplasm was introduced starting in the 1950s, the simplest explanation is that cross-hybridization occurred prior to the collections. All of these arguments need verification.

### Higher genetic diversity in landraces

We compared nucleotide diversity among the groups of accessions based on their seed source and we observed that the landraces were genetically more diverse than the other two groups (elite and advanced lines). There is a greater chance that these landraces possess some valuable alleles associated with biotic and abiotic stress tolerance. Compared to modern elite cultivars, landraces typically exhibit higher genetic diversity [39]. The genetic diversity of the landraces is basically shaped by the activities of farmers, environmental factors and also due to evolutionary forces including random mutations, gene flow between populations and genetic drift [54]. Contrary to this, the selection pressure, rate of recombination and some segregation distortion in modern cultivars during cultivar improvement lead to the loss of certain genes resulting in low genetic diversity [20, 39, 55]. In addition, landraces are subjected to less severe selection compared to the modern elite germplasm and thus, there is a greater chance for maintenance of higher genetic diversity [56]. The landraces harbor some valuable genes associated with adaptation to different stresses as they have been grown under extremely low input environments for years allowing natural selection for alleles associated with adaptive traits [20]. For example, high genetic divergence in a Chinese wheat landrace population was observed due to environmental stresses and individual selection efforts [57]. Landraces promote the introduction of new genetic variation from distant relatives to enable crop improvement in the long run [55] including for stress-tolerance related traits such as drought [58]. For example, the group of Creole wheat landraces (the landraces which were introduced from Europe to Mexico) has been utilized as a source of alleles for different abiotic stresses including drought [59]. Although the current result did not show the presence of a high level of rare alleles (Tajima’s D), the landraces could have been adapted and selected for some specific traits relevant to Nepali wheat growing environments.

### The D genome is the least polymorphic genome as expected

The application of high density SNP markers is now widely used to assess genetic diversity, population structure, and various evolutionary questions [60]. In this study, we identified 95,388 SNP markers across the three wheat genomes. The highest number of polymorphic markers (~ 51%) was found in the B genome similar to a recent study on population of 230 wheat accessions [61]. Furthermore, the highest number of SNPs in the B genome was observed in different synthetic hexaploid wheat (SHWs) populations [14, 43, 62]. The highest number of SNPs was found on chromosome 2B followed by chromosomes 7B and 6B. This is also in accordance with other studies performed on different wheat populations [14, 63]. The proportion of D genome SNPs (~ 10%) observed was low but in agreement with an earlier study [64]. The low level of polymorphism within the D genome could be due to the proposed genetic bottleneck that occurred upon the hybridization of the donor D genome (i.e. *Ae. tauschii*) into the hexaploid genome compared to that of the tetraploid wheat ancestor (AABB) [65]. In other words, hexaploid wheat contains a lower percentage of the diversity present in the wild *Ae. tauschii* genome compared to the wild A and B genomes. An alternative explanation is that the higher proportion of rare alleles in the D genome compared to the A and B genomes may have been more susceptible to genetic drift during modern cultivar development, creating a recent genetic bottleneck in the D genome [66]. The relative contributions of the different genomes to SNP diversity as obtained using GBS-derived SNP markers are also in agreement with the results of past studies performed on hexaploid wheat using DArT markers [14, 67, 68] and SSR markers [34, 38, 41, 69].

### Variation in linkage disequilibrium (LD) patterns may indicate the level of selection pressure

The LD decay distance can indicate the rate of recombination which determines the precision of association and QTL mapping [35, 44, 69]. Various factors affect the random assortment of alleles in a population leading to variation in LD patterns including selection, non-random mating, mutation, admixtures, population size, and genetic drift [70, 71]. Similarly, the type and number of markers also affect LD measurements [35], i.e., limited number of markers result in limited resolution of LD distribution across the genome [42]. More recently, large sets of SNP markers have been used to generate high-resolution maps that result in precise estimations of LD to facilitate the exploitation of genetic resources [44]. In this study, the estimated LD distances for the whole population and the subpopulations indicate shorter LD decay blocks compared to a recent study [40] that demonstrated that the LD decayed at a distance of approximately 1 Mb in a population of 322 soft red winter wheat which is much higher than what we observed in this study despite the population sizes being approximately similar. This result could be due to the presence of higher genetic diversity in the Nepali wheat germplasm. Furthermore, we observed lower LD and faster LD decay for the whole study population compared to the subpopulations, certainly due to the larger size of the former [72]; smaller populations usually have higher LD [14, 44]. Consistent with this observation, the LD for subpopulation 1 (the smallest with only 22 accessions) was the largest despite ~ 50% of the accessions in this group being landraces. As observed by [73], landraces have higher allelic diversity, and the LD decay distance is shorter, compared to elite lines. Here, subpopulation 3 had higher LD and slow LD decay compared to subpopulation 2 despite having a higher proportion of landraces (~ 71%) in the group. Here, the variation in the extent of LD values and LD decay may also be due to genetic drift and/or selection pressure employed on the genetic materials [36, 71, 74] especially CIMMYT lines and Nepali released varieties which form ~ 50% of the population.

## Conclusions and future perspectives

This study provides a novel understanding of the genetic diversity of spring wheat in Nepal. In particular, the relatedness of many accessions regarded as landraces with CIMMYT advanced lines has been a surprising result. The finding suggests that the introduction of genetic resources from the 1950s to recent years significantly altered the population structure of what is currently labeled as native Nepali spring wheat through cross-hybridization and/or collection error. The information generated in this study, including genetic diversity, population structure, and LD, can guide future breeding programs in Nepal. In particular, as more than 42% of the agricultural land in Nepal is rainfed [9] and drought alone contributes 20–30% of yield loss [75], which is expected to worsen due to climate change [76, 77], Nepal is prioritizing the development of new stress tolerant wheat varieties which will be dependent on exploiting germplasm diversity. In this context, this study has shown that only a subset of the wheat “landraces” in Nepal are actually authentic. Utilization of these authentic landraces could be a way forward to utilize useful adaptive traits to improve the future food security of this climate vulnerable nation. Furthermore, utilization of these genetic resources may potentially contribute to global wheat breeding efforts that aim to reduce food insecurity caused by various factors including climate change.

## Methods

### Plant materials

A panel of 318 spring wheat accessions from different sources was assembled for the study, and it was named the Nepali Wheat Diversity Panel (NWDP) (Additional file 1: Table S1). The panel includes 166 Nepali landraces, which were provided by the National Agricultural Genetic Resources Centre (NAGRC) of Nepal. The International Maize and Wheat Improvement Center (CIMMYT, Mexico) contributed 115 advanced breeding lines: these lines were selected based on performance (including diseases and grain yield) following 3 years of field testing (2011–12 season to the 2013–14 seasons) in Nepal. Since the CIMMYT-bred germplasm contributes significantly to the pool of Nepali improved varieties, we included these advanced breeding lines in the NWDP. The National Wheat Research Program (NWRP), NARC, Nepal, also contributed 34 varieties released in Nepal for commercial cultivation until 2014. The objective was to make the diversity panel representative of spring wheat genetic resources available in Nepal. Out of interest, three Canadian spring wheat genotypes were also included in the panel, available from the wheat breeding laboratory, University of Guelph, Canada.

### DNA extraction and genotyping-by-sequencing (GBS)

Genomic DNA of each wheat accession was extracted from leaf tissues collected in the field, using DNeasy Plant Mini Kits (Qiagen, Hilden, Germany) according to the manufacturer’s protocol. SNP genotyping was performed using a GBS approach, and *Pst*I/*Msp*I libraries were prepared as per Poland et al. (2012). Single-end sequencing of multiplex GBS libraries (two PI chips per 96-plex GBS library) was performed on an Ion Proton sequencer at the Plateforme d’Analyses Génomiques [Institut de Biologie Intégrative et des Systèmes (IBIS), Université Laval (Quebec, QC, Canada)].

### GBS data analysis

Ion Torrent sequence reads (50–160 bp) were processed using the Fast-GBS pipeline [27]. In brief, FASTQ files were demultiplexed based on barcode sequences. Demultiplexed reads were trimmed and then mapped against the wheat reference genome [78]. This was followed by the identification of nucleotide variants from mapped reads. Then, variants were removed if they met any of the following criteria: (i) they had more than two alleles; (ii) the overall read quality (QUAL) score was < 32; (iii) the mapping quality (MQ) score was < 30; (iv) read depth was < 2; (v) heterozygosity was > 50%; and the missing data was > 80%. Missing data imputation was performed with BEAGLE v4.1 [79] as described by [80].

### Analysis of SNP distribution and genetic diversity

The distribution of SNPs in the genome was visualized and analyzed by using TASSEL v.5.2.48 [81]. Genetic diversity was characterized by estimating nucleotide diversity (Pi) and divergence (Tajima’s D) using VCFtools 0.1.12b [82]. For these analyses, we used SNPs with a minor allele frequency (MAF) of ≥0.01 and in a window size of 1000 bp. The average Pi and Tajima’s D across all windows were computed to obtain a genome-wide average for each source population.

### Population structure analysis

The model-based Bayesian clustering software fastSTRUCTURE v 1.0 [83] was used to infer the number of subpopulations in the study panel. In order to test the number of subpopulations (K), fastSTRUCTURE was run using the default settings with 100-fold cross-validation, varying the value of K from K = 1 to 10 on 318 accessions. The number of subpopulations that maximized the marginal likelihood was selected using a Python script included in the fastSTRUCTURE package. The structure plot was ordered based on the Q-values generated by fastSTRUCTURE.

In addition, TASSEL v.5.2.48 [81] was used to generate principal components (PCs) from the same SNP data using the covariance method. The proportion of variation explained by each PC was determined by the eigenvalues estimated in the program. Then the PC plots were created using only the first three PCs. Colour coding of the accessions was based on the estimated population structure computed using fastSTRUCTURE.

TASSEL v.5.2.48 [81] was also used to estimate the marker-based genetic distance matrix for all pair-wise combinations using data from 95,388 markers. Hierarchical cluster analysis was performed in R [84] by using the *hclust* function [85]. The unweighted Pair Group Method with Arithmetic Mean (UPGMA) was used to conduct the clustering. Dendrograms were produced using the *as.dendrogram* function, and customization of the dendrograms was performed with the *dendextend* [86] and *circlize* packages in R [87]. Finally, colour coding given to each accession was based on the estimated population structure computed using fastSTRUCTURE.

### Linkage disequilibrium decay analysis

To study linkage disequilibrium (LD) decay in the study population, squared allele frequency correlations (*r*^*2*^) were obtained by using 1000 permutations with comparison-wise significance in TASSEL v.5.2.48 [81]. Then, LD decay was plotted as the relationship between *r*^*2*^ values and the physical distance of the SNP markers in the genomes. LD decay was measured both in the whole population and in the four sub-populations derived from population structure analysis.

## Supplementary Information

**Additional file 1 Table S1.** List of the accessions in the Nepali Wheat Diversity Panel included in the study. [Note: 1) A total of 49 landraces in red text indicate the authentic landraces identified in this study 2) *Rht* genes in specific trait (s) column correspond to presence *Rht1* and/or *Rht2* genes only].

**Additional file 2 Table S2.** Summary of distribution of SNPs in wheat genomes across 21 chromosomes in the Nepali Wheat Diversity Panel.

**Additional file 3 Table S3.** Assignment of individual accession in the Nepali Wheat Diversity Panel to different subpopulation based on Q-matrix obtained from fastSTRUCTURE.

**Additional file 4 Table S4.** The frequency of genotypes in the Nepali Wheat Diversity Panel as differentiated into different subpopulations based on Q-matrix obtained from fastSTRUCTURE.

## Data Availability

The genotypic data (95 K GBS-SNPs) and population structure datasets produced in this study are publicly available at figshare: https://figshare.com/projects/Population_Structure_of_Nepali_Spring_Wheat_Germplasm/90923

## Abbreviations

CIMMYT: International Maize and Wheat Improvement Center
GBS: Genotyping-by-sequencing
LD: Linkage disequilibrium
NARC: Nepal Agriculture Research Council
NAGRC: National Agricultural Genetic Resources Centre
NWDP: Nepali Wheat Diversity Panel
NWRP: National Wheat Research Programme
SNP: Single nucleotide polymorphism
UPGMA: Unweighted pair group method with arithmetic mean
