# Supplementary material for: Population structure of Nepali spring wheat (Triticum aestivum L.) germplasm
Source: BMC Plant Biol. 2020 Nov 23;20:530. doi: 10.1186/s12870-020-02722-8 (PMC7682013; doi:10.1186/s12870-020-02722-8)
Supplement: Supplementary file 1 — Additional file 1 Table S1. List of the accessions in the Nepali Wheat Diversity Panel included in the study. [Note: 1) A total of 49 landraces in red text indicate the authentic landraces identified in this study 2) Rht genes in specific trait (s) column correspond to presence Rht1 and/or Rht2 genes only]. [file 12870_2020_2722_MOESM1_ESM.pdf]

Manuscript title:
Population Structure of Nepali Spring Wheat (*Triticum aestivum* L.) Germplasm

Journal:
BMC Plant Biology

Authors:
*Kamal Khadka[1], Davoud Torkamaneh[1,2,3], Mina Kaviani[1], Francois Belzile[2,3], Manish N. Raizada[1,] and Alireza Navabi[1]

Affiliation:
*[1] Department of Plant Agriculture, University of Guelph, Guelph, Ontario, Canada, N1G 2W1

Corresponding author email address:
kamal.khadka011@gmail.com

Additional file 1: Table S1: List of the genotypes in Nepali Wheat Diversity Panel included in the study [Note: 1) A total of 49 landraces in red text indicate the authentic landraces identified in this study 2) *Rht* genes in specific trait (s) column correspond to presence *Rht1* and/or *Rht2* genes only]

Entry	ID_Name	Pedigree	Seed source	Source/origin	Specific trait(s)
1	NGRC 00176	Local landrace	NAGRC Nepal	Achham district, Nepal	Awnless spike, no *Rht* genes
2	NGRC 00177	Local landrace	NAGRC Nepal	Achham district, Nepal	Awnless spike, no *Rht* genes
3	NGRC 00179	Local landrace	NAGRC Nepal	Achham district, Nepal	Awned spike, no *Rht* genes
4	NGRC 00180	Local landrace	NAGRC Nepal	Achham district, Nepal	Waxy leaves, awnless spike, no *Rht* genes
5	NGRC 00181	Local landrace	NAGRC Nepal	Bajura district, Nepal	Awnless spike, no *Rht* genes
6	NGRC 00199	Local landrace	NAGRC Nepal	Gulmi district, Nepal	Awnless spike, no *Rht* genes
7	NGRC 00202	Local landrace	NAGRC Nepal	Baitadi district, Nepal	Awned spike, no *Rht* genes
8	NGRC 00204	Local landrace	NAGRC Nepal	Dandeldhura district, Nepal	Awnless spike, no *Rht* genes

9	NGRC 00205	Local landrace	NAGRC Nepal	Dandeldhura district, Nepal	Awnless spike, no *Rht* genes
10	NGRC 02448	Local landrace	NAGRC Nepal	Dandeldhura district, Nepal	Awnless spike, no *Rht* genes
11	NGRC 02449	Local landrace	NAGRC Nepal	Baglung district, Nepal	Awnless spike, waxy leaves, no *Rht* genes
12	NGRC 02450	Local landrace	NAGRC Nepal	Baglung district, Nepal	Awned spike, *Rht2* gene
13	NGRC 02451	Local landrace	NAGRC Nepal	Gorkha district, Nepal	Awned spike, waxy leaves, *Rht2* gene
14	NGRC 02452	Local landrace	NAGRC Nepal	Solukhumbu district, Nepal	Awnless spike, no *Rht* genes
15	NGRC 02455	Local landrace	NAGRC Nepal	Solukhumbu district, Nepal	Awnless spike, waxy leaves, no *Rht* genes
16	NGRC 02456	Local landrace	NAGRC Nepal	Khotang district, Nepal	Awned spike, no *Rht* genes
17	NGRC 02457	Local landrace	NAGRC Nepal	Baglung district, Nepal	Awned spike, waxy leaves, *Rht2* gene
18	NGRC 02458	Local landrace	NAGRC Nepal	Myagdi district, Nepal	Awned spike, waxy leaves, no *Rht* genes
19	NGRC 02459	Local landrace	NAGRC Nepal	Mustang district, Nepal	Awned spike, waxy leaves, *Rht2* gene
20	NGRC 02460	Local landrace	NAGRC Nepal	Manang district, Nepal	Awnless spike, no *Rht* genes
21	NGRC 02461	Local landrace	NAGRC Nepal	Manang district, Nepal	Awned spike, waxy leaves, no *Rht* genes
22	NGRC 02462	Local landrace	NAGRC Nepal	Manang district, Nepal	Awnless spike, waxy leaves, no *Rht* genes

23	NGRC 02465	Local landrace	NAGRC Nepal	Mugu district, Nepal	Awnless spike, no *Rht* genes
24	NGRC 02466	Local landrace	NAGRC Nepal	Mugu district, Nepal	Awnless spike, no *Rht* genes
25	NGRC 02467	Local landrace	NAGRC Nepal	Kalikot district, Nepal	Awnless spike, no *Rht* genes
26	NGRC 02470	Local landrace	NAGRC Nepal	Dandeldhura district, Nepal	Awnless spike, no *Rht* genes
27	NGRC 02471	Local landrace	NAGRC Nepal	Jumla district, Nepal	Awnless spike, no *Rht* genes
28	NGRC 02472	Local landrace	NAGRC Nepal	Darchula district, Nepal	Awned spike, waxy leaves, *Rht2* gene
29	NGRC 02496	Local landrace	NAGRC Nepal	Darchula district, Nepal	Awnless spike, no *Rht* genes
30	NGRC 02544	Local landrace	NAGRC Nepal	Rolpa district, Nepal	Awnless spike, waxy leaves, no *Rht* genes
31	NGRC 02546	Local landrace	NAGRC Nepal	Sallyan district, Nepal	Awnless spike, waxy leaves, *Rht1* gene
32	NGRC 02547	Local landrace	NAGRC Nepal	Rukum district, Nepal	Awnless spike, no *Rht* genes
33	NGRC 02548	Local landrace	NAGRC Nepal	Sallyan district, Nepal	Awnless spike, no *Rht* genes
34	NGRC 02549	Local landrace	NAGRC Nepal	Rukum district, Nepal	Awnless spike, no *Rht* genes
35	NGRC 02550	Local landrace	NAGRC Nepal	Sallyan district, Nepal	Awned spike, waxy leaves, *Rht2* gene
36	NGRC 02551	Local landrace	NAGRC Nepal	Sallyan district, Nepal	Awnless spike, no *Rht* genes

37	NGRC 02552	Local landrace	NAGRC Nepal	Sallyan district, Nepal	Awnless spike, no *Rht* genes
38	NGRC 02553	Local landrace	NAGRC Nepal	Rukum district, Nepal	Awnless spike, no *Rht* genes
39	NGRC 02554	Local landrace	NAGRC Nepal	Bajhang district, Nepal	Awned spike, waxy leaves, *Rht*2 gene
40	NGRC 02556	Local landrace	NAGRC Nepal	Mustang district, Nepal	Awnless spike, no *Rht* genes
41	NGRC 02557	Local landrace	NAGRC Nepal	Mustang district, Nepal	Awnless spike, no *Rht* genes
42	NGRC 02558	Local landrace	NAGRC Nepal	Mustang district, Nepal	Awnless spike, no *Rht* genes
43	NGRC 02559	Local landrace	NAGRC Nepal	Mustang district, Nepal	Awnless spike, no *Rht* genes
44	NGRC 02560	Local landrace	NAGRC Nepal	Baglung district, Nepal	Awnless spike, no *Rht* genes
45	NGRC 02561	Local landrace	NAGRC Nepal	Baitadi district, Nepal	Awnless spike, waxy leaves, no *Rht* genes
46	NGRC 02563	Local landrace	NAGRC Nepal	Darchula district, Nepal	Awned spike, waxy leaves, *Rht1* gene
47	NGRC 02564	Local landrace	NAGRC Nepal	Darchula district, Nepal	Awned spike, waxy leaves, *Rht1* gene
48	NGRC 02565	Local landrace	NAGRC Nepal	Bajura district, Nepal	Awnless spike, no *Rht* genes
49	NGRC 02566	Local landrace	NAGRC Nepal	Bajura district, Nepal	Awned spike, no *Rht* genes
50	NGRC 02567	Local landrace	NAGRC Nepal	Bajura district, Nepal	Awned spike, no *Rht* genes

51	NGRC 02568	Local landrace	NAGRC Nepal	Bajura district, Nepal	Awned spike, no *Rht* genes
52	NGRC 02569	Local landrace	NAGRC Nepal	Bajura district, Nepal	Awned spike, no *Rht* genes
53	NGRC 02570	Local landrace	NAGRC Nepal	Bajhang district, Nepal	Awnless spike, no *Rht* genes
54	NGRC 02571	Local landrace	NAGRC Nepal	Bajhang district, Nepal	Awnless spike, no *Rht* genes
55	NGRC 02572	Local landrace	NAGRC Nepal	Bajhang district, Nepal	Awnless spike, no *Rht* genes
56	NGRC 02574	Local landrace	NAGRC Nepal	Jajarkot district, Nepal	Awnless spike, no *Rht genes*
57	NGRC 02575	Local landrace	NAGRC Nepal	Dandeldhura district, Nepal	Awnless spike, no *Rht* genes
58	NGRC 02576	Local landrace	NAGRC Nepal	Dandeldhura district, Nepal	Awnless spike, waxy leaves, no *Rht* genes
59	NGRC 02578	Local landrace	NAGRC Nepal	Dandeldhura district, Nepal	Awned spike, no *Rht* genes
60	NGRC 02579	Local landrace	NAGRC Nepal	Dandeldhura district, Nepal	Awnless spike, no *Rht* genes
61	NGRC 02580	Local landrace	NAGRC Nepal	Dandeldhura district, Nepal	Awnless spike, no *Rht* genes
62	NGRC 02581	Local landrace	NAGRC Nepal	Dandeldhura district, Nepal	Awnless spike, no *Rht* genes
63	NGRC 02582	Local landrace	NAGRC Nepal	Dandeldhura district, Nepal	Awnless spike, no *Rht* genes
64	NGRC 02584	Local landrace	NAGRC Nepal	Taplejung district, Nepal	Awned spike, waxy leaves, *Rht2* gene

65	NGRC 02585	Local landrace	NAGRC Nepal	Doti district, Nepal	Awned spike, waxy leaves, *Rht1* gene
66	NGRC 02586	Local landrace	NAGRC Nepal	Baitadi district, Nepal	Awnless spike, no *Rht* genes
67	NGRC 02587	Local landrace	NAGRC Nepal	Baitadi district, Nepal	Awned spike, waxy leaves, *Rht1* and *Rht2* gene
68	NGRC 02589	Local landrace	NAGRC Nepal	Baitadi district, Nepal	Awned spike, waxy leaves, *Rht1* gene
69	NGRC 02590	Local landrace	NAGRC Nepal	Baitadi district, Nepal	Awnless spike, no *Rht* genes
70	NGRC 02591	Local landrace	NAGRC Nepal	Darchula district, Nepal	Awnless spike, no *Rht* genes
71	NGRC 02593	Local landrace	NAGRC Nepal	Dandeldhura district, Nepal	Awnless spike, waxy leaves, no *Rht* genes
72	NGRC 02594	Local landrace	NAGRC Nepal	Dandeldhura district, Nepal	Awnless spike, no *Rht* genes
73	NGRC 02595	Local landrace	NAGRC Nepal	Doti district, Nepal	Awnless spike, waxy leaves, no *Rht* gene
74	NGRC 02596	Local landrace	NAGRC Nepal	Doti district, Nepal	Awned spike, waxy leaves, *Rht1* gene
75	NGRC 02599	Local landrace	NAGRC Nepal	Dolakha district, Nepal	Awnless spike, no *Rht* genes
76	NGRC 02602	Local landrace	NAGRC Nepal	Kanchanpur district, Nepal	Awned spike, waxy leaves, *Rht1* gene
77	NGRC 02603	Local landrace	NAGRC Nepal	Doti district, Nepal	Awnless spike, no *Rht* genes

78	NGRC 02604	Local landrace	NAGRC Nepal	Dadeldhura district, Nepal	Awnless spike, no *Rht* genes
79	NGRC 02605	Local landrace	NAGRC Nepal	Baitadi district, Nepal	Awnless spike, no *Rht* genes
80	NGRC 02606	Local landrace	NAGRC Nepal	Baitadi district, Nepal	Awnless spike, no *Rht* genes
81	NGRC 02607	Local landrace	NAGRC Nepal	Baitadi district, Nepal	Awnless spike, no *Rht* genes
82	NGRC 02608	Local landrace	NAGRC Nepal	Dadeldhura district, Nepal	Awnless spike, *Rht1* gene
83	NGRC 02609	Local landrace	NAGRC Nepal	Baitadi district, Nepal	Awnless spike, no *Rht* genes
84	NGRC 02610	Local landrace	NAGRC Nepal	Dolkha district, Nepal	Awned spike, waxy leaves, no *Rht* genes
85	NGRC 02611	Local landrace	NAGRC Nepal	Dolkha district, Nepal	Awnless spike, no *Rht* genes
86	NGRC 02612	Local landrace	NAGRC Nepal	Dolkha district, Nepal	Awnless spike, no *Rht* genes
87	NGRC 02613	Local landrace	NAGRC Nepal	Dolkha district, Nepal	Awnless spike, no *Rht* genes
88	NGRC 02614	Local landrace	NAGRC Nepal	Dolkha district, Nepal	Awnless spike, no *Rht* genes
89	NGRC 02615	Local landrace	NAGRC Nepal	Dolkha district, Nepal	Awnless spike, no *Rht* genes
90	NGRC 02617	Local landrace	NAGRC Nepal	Dolkha district, Nepal	Awnless spike, no *Rht* genes
91	NGRC 02619	Local landrace	NAGRC Nepal	Dolkha district, Nepal	Awned spike, no *Rht* genes

92	NGRC 02620	Local landrace	NAGRC Nepal	Dolkha district, Nepal	Awnless spike, no *Rht* genes
93	NGRC 02621	Local landrace	NAGRC Nepal	Rasuwa district, Nepal	Awned spike, *Rht2* gene
94	NGRC 02622	Local landrace	NAGRC Nepal	Dolkha district, Nepal	Awnless spike, waxy leaves, no *Rht* genes
95	NGRC 02623	Local landrace	NAGRC Nepal	Dolkha district, Nepal	Awnless spike, no *Rht* genes
96	NGRC 02624	Local landrace	NAGRC Nepal	Dolpa district, Nepal	Awnless spike, no *Rht* genes
97	NGRC 02625	Local landrace	NAGRC Nepal	Dolkha district, Nepal	Awnless spike, no *Rht* genes
98	NGRC 02629	Local landrace	NAGRC Nepal	Dolkha district, Nepal	Awned spike, no *Rht* genes
99	NGRC 02630	Local landrace	NAGRC Nepal	Dolkha district, Nepal	Awnless spike, no *Rht* genes
100	NGRC 02631	Local landrace	NAGRC Nepal	Rasuwa district, Nepal	Awned spike, waxy leaves, no *Rht* genes
101	NGRC 02632	Local landrace	NAGRC Nepal	Nuwakot district, Nepal	Awned spike, waxy leaves, no *Rht* genes
102	NGRC 02633	Local landrace	NAGRC Nepal	Nuwakot district, Nepal	Awned spike, *Rht2* gene
103	NGRC 04399	Local landrace	NAGRC Nepal	Ramechhap district, Nepal	Awnless spike, no *Rht* genes
104	NGRC 04400	Local landrace	NAGRC Nepal	Ramechhap district, Nepal	Awned spike, waxy leaves, *Rht1* gene
105	NGRC 04401	Local landrace	NAGRC Nepal	Ramechhap district, Nepal	Awned spike, waxy leaves, *Rht1* gene

106	NGRC 04402	Local landrace	NAGRC Nepal	Ramechhap district, Nepal	Awnless spike, no *Rht* genes
107	NGRC 04404	Local landrace	NAGRC Nepal	Ramechhap district, Nepal	Awned spike, *Rht2* gene
108	NGRC 04405	Local landrace	NAGRC Nepal	Ramechhap district, Nepal	Awnless spike, waxy leaves, no *Rht* genes
109	NGRC 04406	Local landrace	NAGRC Nepal	Ramechhap district, Nepal	Awnless spike, no *Rht* gene
110	NGRC 04408	Local landrace	NAGRC Nepal	Ramechhap district, Nepal	Awnless spike, no *Rht* genes
111	NGRC 04409	Local landrace	NAGRC Nepal	Salyan district, Nepal	Awnless spike, no *Rht* genes
112	NGRC 04410	Local landrace	NAGRC Nepal	Myagdi district, Nepal	Awned spike, no *Rht* genes
113	NGRC 04413	Local landrace	NAGRC Nepal	Kavreplanchok district, Nepal	Awnless spike, no *Rht* genes
114	NGRC 04414	Local landrace	NAGRC Nepal	Kavreplanchok district, Nepal	Awned spike, waxy leaves, *Rht1* gene
115	NGRC 04416	Local landrace	NAGRC Nepal	Salyan district, Nepal	Awnless spike, no *Rht* genes
116	NGRC 04417	Local landrace	NAGRC Nepal	Salyan district, Nepal	Awnless spike, no *Rht* genes
117	NGRC 04418	Local landrace	NAGRC Nepal	Salyan district, Nepal	Awnless spike, waxy leaves, no *Rht* genes
118	NGRC 04419	Local landrace	NAGRC Nepal	Salyan district, Nepal	Awnless spike, waxy leaves, no *Rht* genes
119	NGRC 04421	Local landrace	NAGRC Nepal	Salyan district, Nepal	Awnless spike, no *Rht* genes

120	NGRC 04422	Local landrace	NAGRC Nepal	Surkhet district, Nepal	Awnless spike, waxy leaves, no *Rht* genes
121	NGRC 04423	Local landrace	NAGRC Nepal	Surkhet district, Nepal	Awnless spike, waxy leaves, no *Rht* genes
122	NGRC 04424	Local landrace	NAGRC Nepal	Ramechhap district, Nepal	Awnless spike, waxy leaves, no *Rht* genes
123	NGRC 04425	Local landrace	NAGRC Nepal	Surkhet district, Nepal	Awnless spike, waxy leaves, no *Rht* genes
124	NGRC 04426	Local landrace	NAGRC Nepal	Surkhet district, Nepal	Awnless spike, waxy leaves, no *Rht* genes
125	NGRC 04427	Local landrace	NAGRC Nepal	Surkhet district, Nepal	Awned spike, *Rht2* gene
126	NGRC 04428	Local landrace	NAGRC Nepal	Dolakha district, Nepal	Awnless spike, no *Rht* genes
127	NGRC 04429	Local landrace	NAGRC Nepal	Dolakha district, Nepal	Awnless spike, no *Rht* genes
128	NGRC 04430	Local landrace	NAGRC Nepal	Dolakha district, Nepal	Awnless spike, no *Rht* genes
129	NGRC 04431	Local landrace	NAGRC Nepal	Dolakha district, Nepal	Awnless spike, no *Rht* genes
130	NGRC 04432	Local landrace	NAGRC Nepal	Dolakha district, Nepal	Awnless spike, no *Rht* genes
131	NGRC 04433	Local landrace	NAGRC Nepal	Dolakha district, Nepal	Awnless spike, no *Rht* genes
132	NGRC 04434	Local landrace	NAGRC Nepal	Dolakha district, Nepal	Awnless spike, no *Rht* genes
133	NGRC 04436	Local landrace	NAGRC Nepal	Dolakha district, Nepal	Awned spike, no *Rht* genes

134	NGRC 04437	Local landrace	NAGRC Nepal	Dolakha district, Nepal	Awned spike, waxy leaves, *Rht1* gene
135	NGRC 04439	Local landrace	NAGRC Nepal	Dolakha district, Nepal	Awnless spike, waxy leaves, no *Rht* genes
136	NGRC 04440	Local landrace	NAGRC Nepal	Dolakha district, Nepal	Awnless spike, waxy leaves, no *Rht* genes
137	NGRC 04443	Local landrace	NAGRC Nepal	Kavreplanchok district, Nepal	Awnless spike, *Rht1*and *Rht2* genes
138	NGRC 04444	Local landrace	NAGRC Nepal	Kavreplanchok district, Nepal	Awnless spike, no *Rht* genes
139	NGRC 04445	Local landrace	NAGRC Nepal	Kavreplanchok district, Nepal	Awnless spike, no *Rht* genes
140	NGRC 04446	Local landrace	NAGRC Nepal	Kavreplanchok district, Nepal	Awned spike, no *Rht* genes
141	NGRC 04447	Local landrace	NAGRC Nepal	Kavreplanchok district, Nepal	Awned spike, waxy leaves, *Rht1* gene
142	NGRC 04448	Local landrace	NAGRC Nepal	Surkhet district, Nepal	Awnless spike, no *Rht* genes
143	NGRC 04449	Local landrace	NAGRC Nepal	Surkhet district, Nepal	Awned spike, waxy leaves, *Rht1* gene
144	NGRC 04450	Local landrace	NAGRC Nepal	Surkhet district, Nepal	Awnless spike, waxy leaves
145	NGRC 04451	Local landrace	NAGRC Nepal	Dailekh district, Nepal	Awnless spike, no *Rht* genes
146	NGRC 04452	Local landrace	NAGRC Nepal	Dailekh district, Nepal	Awnless spike, no *Rht* genes
147	NGRC 04453	Local landrace	NAGRC Nepal	Dailekh district, Nepal	Awnless spike, no *Rht* genes

148	NGRC 04454	Local landrace	NAGRC Nepal	Dailekh district, Nepal	Awnless spike, no *Rht* genes
149	NGRC 04455	Local landrace	NAGRC Nepal	Dailekh district, Nepal	Awnless spike, no *Rht* genes
150	NGRC 04456	Local landrace	NAGRC Nepal	Dailekh district, Nepal	Awned spike, waxy leaves, *Rht1* gene
151	NGRC 04457	Local landrace	NAGRC Nepal	Dailekh district, Nepal	Awnless spike, no *Rht* genes
152	NGRC 04458	Local landrace	NAGRC Nepal	Dailekh district, Nepal	Awned spike, waxy leaves, no *Rht1* gene
153	NGRC 04459	Local landrace	NAGRC Nepal	Dailekh district, Nepal	Awnless spike, no *Rht* genes
154	NGRC 04460	Local landrace	NAGRC Nepal	Dailekh district, Nepal	Awnless spike, no *Rht* genes
155	NGRC 04461	Local landrace	NAGRC Nepal	Dailekh district, Nepal	Awnless spike, no *Rht* genes
156	NGRC 04462	Local landrace	NAGRC Nepal	Dailekh district, Nepal	Awnless spike, no *Rht* genes
157	NGRC 04463	Local landrace	NAGRC Nepal	Dailekh district, Nepal	Awned spike, no *Rht* genes
158	NGRC 04464	Local landrace	NAGRC Nepal	Dailekh district, Nepal	Awnless spike, no *Rht* genes
159	NGRC 04465	Local landrace	NAGRC Nepal	Dailekh district, Nepal	Awnless spike, no *Rht* genes
160	NGRC 04466	Local landrace	NAGRC Nepal	Dailekh district, Nepal	Awnless spike, *Rht2* gene
161	NGRC 04467	Local landrace	NAGRC Nepal	Dailekh district, Nepal	Awnless spike, no *Rht* genes

162	NGRC 04468	Local landrace	NAGRC Nepal	Dailekh district, Nepal	Awnless spike, no *Rht* genes
163	NGRC 04470	Local landrace	NAGRC Nepal	Dailekh district, Nepal	Awnless spike, no *Rht* genes
164	NGRC 04471	Local landrace	NAGRC Nepal	Dailekh district, Nepal	Awnless spike, no *Rht* genes
165	NGRC 04472	Local landrace	NAGRC Nepal	Dailekh district, Nepal	Awnless spike, no *Rht* genes
166	NGRC 04473	Local landrace	NAGRC Nepal	Dailekh district, Nepal	Awnless spike, no *Rht* genes
167	NGRC 04474	Local landrace	NAGRC Nepal	Mugu district, Nepal	Awnless spike, no *Rht* genes
168	Lerma 52	MENTANA/KENYA 324	NWRP Nepal (Released variety)	Mexico	Semi-dwarf, awned spike, *Rht1* gene
169	Kalyansona	PJ"S"/GB55 (S227)	NWRP Nepal (Released variety)	Mexico	Semi-dwarf, awned spike, *Rht1* gene
170	Pitic 62	YT54/N10B126.IC	NWRP Nepal (Released variety)	Mexico	Semi-dwarf, awned spike, *Rht2* gene
171	RR21	1154-388/AN/3/YT54/NIOB/RL64	NWRP Nepal (Released variety)	Mexico	Semi-dwarf, awned spike, *Rht2* gene
172	NL 30	HD832-5-5-OY/RB	NWRP Nepal (Released variety)	India	Semi-dwarf, awned spike, *Rht2* gene
173	UP 262	S308/BAJIO-66	NWRP Nepal (Released variety)	India	Semi-dwarf, awned spike, *Rht2* gene
174	Lumbini	E4871/PJ62	NWRP Nepal (Released variety)	India	Semi-dwarf, awned spike, *Rht1* gene
175	Tribeni	HD1963/HD1931	NWRP Nepal (Released variety)	India	Semi-dwarf, awned spike, *Rht1* gene
176	Vinayak	LC55	NWRP Nepal (Released variety)	India	Semi-dwarf, awned spike, *Rht2* gene

177	Siddhartha	HD2092/HD1982//E4870/K65	NWRP Nepal (Released variety)	India	Awned spike, no *Rht* genes
178	Vaskar	TZPP/PL//7C	NWRP Nepal (Released variety)	Mexico	Semi-dwarf, awned spike, *Rht1* gene
179	Nepal 297	HD2173/HD2186//HD2160	NWRP Nepal (Released variety)	India	Semi-dwarf, awned spike, *Rht1* gene
180	NL 251	WH147/HD2160//WH147	NWRP Nepal (Released variety)	India	Semi-dwarf, awned spike, *Rht2* gene
181	Annapurna 1	KBZ/BUHO//KAL/BB=(VEE"S")	NWRP Nepal (Released variety)	Mexico	Dense and awned spike, amber grain, *Rht1* gene
182	Annapurna 2	NPO/TOB"S"//8156/3/KAL/BB	NWRP Nepal (Released variety)	India	Semi-dwarf, awned spike, *Rht1* gene
183	Annapurna 3	KBZ/BUHO//KAL/BB=(VEE"S")	NWRP Nepal (Released variety)	Mexico	Semi-dwarf, awned spike, *Rht1* gene
184	BL 1022	PVN/BUC	NWRP Nepal (Released variety)	Mexico	Semi-dwarf, awned spike, *Rht1* gene
185	Bhrikuti	CMT/COC75/3/PLO//FURY/ANA75	NWRP Nepal (Released variety)	Mexico	Semi-dwarf, awned spike, *Rht1* gene
186	BL 1135	QTZ/TAN"S"	NWRP Nepal (Released variety)	Nepal	Semi-dwarf, awned spike, *Rht2* gene
187	Annapurna 4	KBZ/3/CC/INIA//CNO/ELGAU/SN64	NWRP Nepal (Released variety)	Mexico	Awned spike, no *Rht* genes
188	Achyut	CPAN168/HD2204	NWRP Nepal (Released variety)	India	Semi-dwarf, lower plains and foot hills, awned spike, *Rht1* gene
189	Rohini	PRL"S"/TONI//CHIL"S"	NWRP Nepal (Released variety)	Nepal	Awned spike, no *Rht* genes
190	Kanti	LIRA/FUFAN17//VEE#5"S"	NWRP Nepal (Released variety)	Mexico	Awned spike, no *Rht* genes
191	Pasang Lhamu	PGO/SERI	NWRP Nepal (Released variety)	Mexico	Awned spike, semi erect leaves, bold grains, awned spike, no *Rht* genes
192	BL 1473	NL297/NL352	NWRP Nepal (Released variety)	Nepal	Semi-dwarf, spot blotch tolerant, awned spike, *Rht2* gene

193	Gautam	SIDDHARTH/NING8319/NL297	NWRP Nepal (Released variety)	Nepal	Semi-dwarf, high yielding, awned spike, *Rht2* gene
194	WK1204	SW89-3064/STAR	NWRP Nepal (Released variety)	Mexico	Semi-erect waxy leaves, sturdy stem, awned and waxy spike, *Rht2* gene
195	NL 971	MRNG/BUC//BLO/PVN/3/PJB81	NWRP Nepal (Released variety)	Mexico	Semi-dwarf, high yielding, white grains awned spike, *Rht1* gene
196	Aditya (BL 3264)	GS348/NL746//NL748	NWRP Nepal (Released variety)	Nepal	White grain, high yielding, awned spike, *Rht2* gene
197	Vijay (BL 3063)	NL 748/NL 736 (Ug99 Res.)	NWRP Nepal (Released variety)	Nepal	Semi-dwarf, terminal heat tolerance, awned spike, *Rht2* gene
198	Gaura (BL 3235)	NL 872/NL 868	NWRP Nepal (Released variety)	Nepal	Early, spot blotch resitant, high tillering, awned spike, no *Rht* genes
199	Dhaulagiri (BL 3503)	BL 1961/NL 867	NWRP Nepal (Released variety)	Nepal	Awned and waxi spike, erect leaves, *Rht2* gene
200	Danphe (NL 1064)	KIRITATI//2*PBW65/2*SERI.1B	NWRP Nepal (Released variety)	Mexico	Awned spike, waxy and droopy leaves, *Rht1* gene
201	Tilottama (NL 1073)	WAXWING*2/VIVITSI	NWRP Nepal (Released variety)	Mexico	Early, stay green, spot blotch resistant, awned spike, *Rht1* gene
202	BW30655	PBW343	CIMMYT, Mexico (CIMMYT line)	CIMMYT, Mexico	Semi-dwarf, awned spike, waxy leaves, *Rht1* gene
203	BW35623	PRL/2*PASTOR	CIMMYT, Mexico (CIMMYT line)	CIMMYT, Mexico	Semi-dwarf, awned spike, waxy leaves, *Rht1* gene
204	BW43945	MUNAL #1	CIMMYT, Mexico (CIMMYT line)	CIMMYT, Mexico	Semi-dwarf, awned spike, waxy leaves, *Rht1* gene

205	BW45161	BECARD	CIMMYT, Mexico (CIMMYT line)	CIMMYT, Mexico	Semi-dwarf, awned spike, waxy leaves, *Rht1* gene
206	BW43354	SUPER 152	CIMMYT, Mexico (CIMMYT line)	CIMMYT, Mexico	Semi-dwarf, awned spike, waxy leaves, *Rht1* gene
207	BW44829	PFUNYE #1	CIMMYT, Mexico (CIMMYT line)	CIMMYT, Mexico	Semi-dwarf, awned spike, waxy leaves, *Rht1* gene
208	BW44908	HUHWA	CIMMYT, Mexico (CIMMYT line)	CIMMYT, Mexico	Semi-dwarf, awned spike, waxy leaves, *Rht1* gene
209	BW45568	TRCH*2/3/C80.1/3*QT4118//3*PASTOR	CIMMYT, Mexico (CIMMYT line)	CIMMYT, Mexico	Semi-dwarf, awned spike, waxy leaves, *Rht1* gene
210	BW45152	WHEAR//INQALAB 91*2/TUKURU	CIMMYT, Mexico (CIMMYT line)	CIMMYT, Mexico	Awnless spike, waxy leaves, no *Rht* genes
211	BW45573	WHEAR/KRONSTAD F2004	CIMMYT, Mexico (CIMMYT line)	CIMMYT, Mexico	Awnless spike, waxy leaves, no *Rht* genes
212	BW45173	WHEAR/SOKOLL	CIMMYT, Mexico (CIMMYT line)	CIMMYT, Mexico	Semi-dwarf, awned spike, waxy leaves, *Rht1* gene
213	BW45578	SHA7/VEE#5/5/VEE#8//JUP/BJY/3/F3.71/TRM/4/2*WEAVER/6/SKAUZ/PARUS//PARUS	CIMMYT, Mexico (CIMMYT line)	CIMMYT, Mexico	Semi-dwarf, awned spike, waxy leaves, *Rht1* gene
214	BW45590	PFAU/SERI.1B//AMAD/3/INQALAB 91*2/KUKUNA/4/WBLL1*2/KURUKU	CIMMYT, Mexico (CIMMYT line)	CIMMYT, Mexico	Semi-dwarf, awned spike, waxy leaves, *Rht1* gene

215	BW45592	NELOKI	CIMMYT, Mexico (CIMMYT line)	CIMMYT, Mexico	Semi-dwarf, awned spike, waxy leaves, *Rht1* gene
216	BW45593	HUW234+LR34/PRINIA//INQALAB 91*2/KUKUNA/5/FRET2*2/4/SNI/TRAP#1/3/KAUZ*2/TRAP//KAUZ	CIMMYT, Mexico (CIMMYT line)	CIMMYT, Mexico	Semi-dwarf, awned spike, waxy leaves, *Rht1* gene
217	BW45587	SNB//CMH79A.955/3*CNO79/3/ATTILA/4/CHEN/AEGILOPS SQUARROSA (TAUS)//BCN/3/2*KAUZ	CIMMYT, Mexico (CIMMYT line)	CIMMYT, Mexico	Semi-dwarf, awned spike, waxy leaves, *Rht1* gene
218	BW45165	KAUZ//ALTAR 84/AOS/3/PASTOR/4/MILAN/CUPE//SW89.3064/5/KIRITATI	CIMMYT, Mexico (CIMMYT line)	CIMMYT, Mexico	Semi-dwarf, awned spike, waxy leaves, *Rht1* gene
219	BW45595	MELON//FILIN/MILAN/3/FILIN	CIMMYT, Mexico (CIMMYT line)	CIMMYT, Mexico	Semi-dwarf, awned spike, waxy leaves, *Rht1* gene
220	BW48132	MARCHOUCH*4/SAADA/3/2*FRET2/KUKUNA//FRET2	CIMMYT, Mexico (CIMMYT line)	CIMMYT, Mexico	Semi-dwarf, awned spike, waxy leaves, *Rht1* gene
221	BW48133	WAXWING/6/PVN//CAR422/ANA/5/BOW/CROW//BUC/PVN/3/YR/4/TRAP#1	CIMMYT, Mexico (CIMMYT line)	CIMMYT, Mexico	Semi-dwarf, awned spike, waxy leaves, *Rht1* gene
222	BW48135	ATTILA*2//CHIL/BUC*2/3/KUKUNA	CIMMYT, Mexico (CIMMYT line)	CIMMYT, Mexico	Semi-dwarf, awned spike, waxy leaves, *Rht1* gene
223	BW48136	WBLL1/KUKUNA//TACUPETO F2001/3/BAJ #1	CIMMYT, Mexico (CIMMYT line)	CIMMYT, Mexico	Semi-dwarf, awned spike, waxy leaves, *Rht1* gene

224	BW48137	WBLL1//UP2338*2/VIVITSI	CIMMYT, Mexico (CIMMYT line)	CIMMYT, Mexico	Semi-dwarf, awned spike, waxy leaves, *Rht1* gene
225	BW48139	FRET2*2/4/SNI/TRAP#1/3/KAUZ*2/TRAP//KAUZ/5/PFAU/WEAVER//BRAMBLING	CIMMYT, Mexico (CIMMYT line)	CIMMYT, Mexico	Semi-dwarf, awned spike, waxy leaves, *Rht2* gene
226	BW48171	TRCH//PRINIA/PASTOR	CIMMYT, Mexico (CIMMYT line)	CIMMYT, Mexico	Semi-dwarf, awned spike, waxy leaves, *Rht1* gene
227	BW48140	KAUZ//ALTAR 84/AOS/3/MILAN/KAUZ/4/SAUAL	CIMMYT, Mexico (CIMMYT line)	CIMMYT, Mexico	Semi-dwarf, awned spike, waxy leaves, *Rht1* gene
228	BW48141	KACHU/SAUAL	CIMMYT, Mexico (CIMMYT line)	CIMMYT, Mexico	Semi-dwarf, awned spike, waxy leaves, *Rht1* gene
229	BW48144	ATTILA/3*BCN//BAV92/3/TILHI/5/BAV92/3/PRL/SARA//TSI/VEE#5/4/CROC_1/AE.SQUARROSA (224)//2*OPATA	CIMMYT, Mexico (CIMMYT line)	CIMMYT, Mexico	Semi-dwarf, awned spike, waxy leaves, *Rht1* gene
230	BW48145	ROLF07/YANAC//TACUPETO F2001/BRAMBLING	CIMMYT, Mexico (CIMMYT line)	CIMMYT, Mexico	Semi-dwarf, awned spike, waxy leaves, *Rht1* gene
231	BW48148	FRET2*2/4/SNI/TRAP#1/3/KAUZ*2/TRAP//KAUZ*2/6/PVN//CAR422/ANA/5/BOW/CROW//BUC/PVN/3/YR/4/TRAP#1	CIMMYT, Mexico (CIMMYT line)	CIMMYT, Mexico	Semi-dwarf, awned spike, waxy leaves, *Rht1* gene
232	BW48149	FRET2*2/4/SNI/TRAP#1/3/KAUZ*2/TRAP//KAUZ/5/PARUS/6/FRET2*2/KUKUNA	CIMMYT, Mexico (CIMMYT line)	CIMMYT, Mexico	Semi-dwarf, awned spike, waxy leaves, *Rht1* gene

233	BW48150	FRET2*2/4/SNI/TRAP#1/3/KAUZ*2/TRAP//KAUZ/5/PARUS/6/FRET2*2/KUKUNA	CIMMYT, Mexico (CIMMYT line)	CIMMYT, Mexico	Semi-dwarf, awned spike, waxy leaves, *Rht1* gene
234	BW48151	FRET2*2/4/SNI/TRAP#1/3/KAUZ*2/TRAP//KAUZ*2/5/KIRITATI	CIMMYT, Mexico (CIMMYT line)	CIMMYT, Mexico	Semi-dwarf, awned spike, waxy leaves, *Rht1* gene
235	BW48154	FRET2/KUKUNA//FRET2/3/PASTOR//HXL7573/2*BAU/5/FRET2*2/4/SNI/TRAP#1/3/KAUZ*2/TRAP//KAUZ	CIMMYT, Mexico (CIMMYT line)	CIMMYT, Mexico	Semi-dwarf, awned spike, waxy leaves, *Rht1* gene
236	BW48155	WBLL1*2/KUKUNA*2//WHEAR	CIMMYT, Mexico (CIMMYT line)	CIMMYT, Mexico	Semi-dwarf, awned spike, waxy leaves, *Rht1* gene
237	BW48156	TRCH/SRTU//KACHU	CIMMYT, Mexico (CIMMYT line)	CIMMYT, Mexico	Semi-dwarf, awned spike, waxy leaves, *Rht1* gene
238	BW48158	SERI.1B//KAUZ/HEVO/3/AMAD*2/4/KIRITATI	CIMMYT, Mexico (CIMMYT line)	CIMMYT, Mexico	Semi-dwarf, awned spike, waxy leaves, *Rht1* gene
239	BW48172	WAXWING*2/6/PVN//CAR422/ANA/5/BOW/CROW//BUC/PVN/3/YR/4/TRAP#1	CIMMYT, Mexico (CIMMYT line)	CIMMYT, Mexico	Semi-dwarf, awned spike, waxy leaves, *Rht1* gene
240	BW48174	PBW343*2/KUKUNA//PARUS/3/PBW343*2/KUKUNA	CIMMYT, Mexico (CIMMYT line)	CIMMYT, Mexico	Semi-dwarf, awned spike, waxy leaves, *Rht1* and *Rht2* genes
241	BW48161	PBW343*2/KUKUNA//PARUS/3/PBW343*2/KUKUNA	CIMMYT, Mexico (CIMMYT line)	CIMMYT, Mexico	Semi-dwarf, awned spike, waxy leaves, *Rht1* gene

242	BW48162	PBW343*2/KUKUNA*2//YANAC	CIMMYT, Mexico (CIMMYT line)	CIMMYT, Mexico	Semi-dwarf, awned spike, waxy leaves, *Rht1* gene
243	BW48163	PBW343*2/KUKUNA*2//YANAC	CIMMYT, Mexico (CIMMYT line)	CIMMYT, Mexico	Semi-dwarf, awned spike, waxy leaves, *Rht1* gene
244	BW48166	PBW343*2/KUKUNA//SRTU/3/PBW343*2/KHVAKI	CIMMYT, Mexico (CIMMYT line)	CIMMYT, Mexico	Semi-dwarf, awned spike, waxy leaves, *Rht1* gene
245	BW48165	PBW343*2/KUKUNA//SRTU/3/PBW343*2/KHVAKI	CIMMYT, Mexico (CIMMYT line)	CIMMYT, Mexico	Semi-dwarf, awned spike, waxy leaves, *Rht1* gene
246	BW48169	ATTILA*2/PBW65/6/PVN//CAR422/ANA/5/BOW/CROW//BUC/PVN/3/YR/4/TRAP#1/7/ATTILA/2*PASTOR	CIMMYT, Mexico (CIMMYT line)	CIMMYT, Mexico	Semi-dwarf, awned spike, waxy leaves, *Rht1* gene
247	BW48168	ATTILA*2/PBW65/6/PVN//CAR422/ANA/5/BOW/CROW//BUC/PVN/3/YR/4/TRAP#1/7/ATTILA/2*PASTOR	CIMMYT, Mexico (CIMMYT line)	CIMMYT, Mexico	Semi-dwarf, awned spike, waxy leaves, *Rht1* gene
248	BW49069	ATTILA*2/PBW65*2//KACHU	CIMMYT, Mexico (CIMMYT line)	CIMMYT, Mexico	Semi-dwarf, awned spike, waxy leaves, *Rht1* gene
249	BW49227	ATTILA*2/PBW65*2//KACHU	CIMMYT, Mexico (CIMMYT line)	CIMMYT, Mexico	Semi-dwarf, awned spike, waxy leaves, *Rht1* gene
250	BW49235	REEDLING #1	CIMMYT, Mexico (CIMMYT line)	CIMMYT, Mexico	Semi-dwarf, awned spike, waxy leaves, *Rht1* gene

251	BW49072	KACHU #1/KIRITATI//KACHU	CIMMYT, Mexico (CIMMYT line)	CIMMYT, Mexico	Semi-dwarf, awned spike, waxy leaves, *Rht1* gene
252	BW49075	PBW343*2/KUKUNA*2//FRTL/PIFED	CIMMYT, Mexico (CIMMYT line)	CIMMYT, Mexico	Semi-dwarf, awned spike, waxy leaves, *Rht1* gene
253	BW49077	WBLL1*2/4/BABAX/LR42//BABAX/3/BABAX/LR42//BABAX	CIMMYT, Mexico (CIMMYT line)	CIMMYT, Mexico	Semi-dwarf, awned spike, waxy leaves, *Rht1* gene
254	BW49078	ATTILA*2/PBW65*2//MURGA	CIMMYT, Mexico (CIMMYT line)	CIMMYT, Mexico	Semi-dwarf, awned spike, waxy leaves, *Rht1* gene
255	BW49079	ROLF07*2/5/REH/HARE//2*BCN/3/CROC_1/AE.SQUARROSA (213)//PGO/4/HUITES	CIMMYT, Mexico (CIMMYT line)	CIMMYT, Mexico	Semi-dwarf, awned spike, waxy leaves, *Rht1* gene
256	BW49082	ROLF07*2/5/FCT/3/GOV/AZ//MUS/4/DOVE/BUC	CIMMYT, Mexico (CIMMYT line)	CIMMYT, Mexico	Semi-dwarf, awned spike, waxy leaves, *Rht1* gene
257	BW49344	SAUAL/3/ACHTAR*3//KANZ/KS85-8-4/4/SAUAL	CIMMYT, Mexico (CIMMYT line)	CIMMYT, Mexico	Semi-dwarf, awned spike, waxy leaves, *Rht1* gene
258	BW49084	FRNCLN/ROLF07	CIMMYT, Mexico (CIMMYT line)	CIMMYT, Mexico	Semi-dwarf, awned spike, waxy leaves, *Rht1* gene
259	BW49088	QUAIU/5/FRET2*2/4/SNI/TRAP#1/3/KAUZ*2/TRAP//KAUZ	CIMMYT, Mexico (CIMMYT line)	CIMMYT, Mexico	Semi-dwarf, awned spike, waxy leaves, *Rht1* gene
260	BW49089	TACUPETO F2001*2/BRAMBLING//WBLL1*2/BRAMBLING	CIMMYT, Mexico (CIMMYT line)	CIMMYT, Mexico	Semi-dwarf, awned spike, waxy leaves, *Rht1* gene

261	BW49391	BECARD/KACHU	CIMMYT, Mexico (CIMMYT line)	CIMMYT, Mexico	Semi-dwarf, awned spike, waxy leaves, *Rht1* gene
262	BW49092	BECARD/KACHU	CIMMYT, Mexico (CIMMYT line)	CIMMYT, Mexico	Semi-dwarf, awned spike, waxy leaves, *Rht1* gene
263	BW49093	BECARD/KACHU	CIMMYT, Mexico (CIMMYT line)	CIMMYT, Mexico	Semi-dwarf, awned spike, waxy leaves, *Rht1* gene
264	BW49399	ALTAR 84/AE.SQUARROSA (221)//3*BORL95/3/URES/JUN//KAUZ /4/WBLL1/5/MUTUS	CIMMYT, Mexico (CIMMYT line)	CIMMYT, Mexico	Semi-dwarf, awned spike, waxy leaves, *Rht1* gene
265	BW49095	TRCH/HUIRIVIS #1	CIMMYT, Mexico (CIMMYT line)	CIMMYT, Mexico	Semi-dwarf, awned spike, waxy leaves, *Rht1* gene
266	BW49097	BECARD/AKURI	CIMMYT, Mexico (CIMMYT line)	CIMMYT, Mexico	Semi-dwarf, awned spike, waxy leaves, *Rht1* gene
267	BW49099	KINGBIRD #1//INQALAB 91*2/TUKURU	CIMMYT, Mexico (CIMMYT line)	CIMMYT, Mexico	Semi-dwarf, awned spike, waxy leaves, *Rht1* gene
268	BW49102	QUELEA	CIMMYT, Mexico (CIMMYT line)	CIMMYT, Mexico	Semi-dwarf, awned spike, waxy leaves, *Rht1* gene
269	BW49108	UP2338*2/VIVITSI/3/FRET2/TUKUR U//FRET2/4/MISR 1	CIMMYT, Mexico (CIMMYT line)	CIMMYT, Mexico	Semi-dwarf, awned spike, waxy leaves, *Rht1* gene
270	BW49109	WAXBILL	CIMMYT, Mexico (CIMMYT line)	CIMMYT, Mexico	Semi-dwarf, awned spike, waxy leaves, *Rht1* gene

271	BW49110	BAV92//IRENA/KAUZ/3/HUITES/4/GONDO/TNMU/5/BAV92//IRENA/KAUZ/3/HUITES	CIMMYT, Mexico (CIMMYT line)	CIMMYT, Mexico	Semi-dwarf, awned spike, waxy leaves, *Rht1* gene
272	BW49111	WBLL1*2/TUKURU//FN/2*PASTOR/3/FRET2/KIRITATI	CIMMYT, Mexico (CIMMYT line)	CIMMYT, Mexico	Semi-dwarf, awned spike, waxy leaves, *Rht1* gene
273	BW49112	PVN/5/2*REH/HARE//2*BCN/3/CROC_1/AE.SQUARROSA (213)//PGO/4/HUITES	CIMMYT, Mexico (CIMMYT line)	CIMMYT, Mexico	Semi-dwarf, awned spike, waxy leaves, *Rht1* gene
274	BW49113	KFA/2*KACHU	CIMMYT, Mexico (CIMMYT line)	CIMMYT, Mexico	Semi-dwarf, awned spike, waxy leaves, *Rht1* gene
275	BW49922	CHIBIA//PRLII/CM65531/3/MISR2, EGY/4/MUNAL #1	CIMMYT, Mexico (CIMMYT line)	CIMMYT, Mexico	Semi-dwarf, awned spike, waxy leaves, *Rht1* gene
276	BW49923	KACHU//WBLL1*2/BRAMBLING	CIMMYT, Mexico (CIMMYT line)	CIMMYT, Mexico	Semi-dwarf, awned spike, waxy leaves, *Rht1* gene
277	BW49924	KACHU/KIRITATI	CIMMYT, Mexico (CIMMYT line)	CIMMYT, Mexico	Semi-dwarf, awned spike, waxy leaves, *Rht1* gene
278	BW49927	SUP152/BAJ #1	CIMMYT, Mexico (CIMMYT line)	CIMMYT, Mexico	Semi-dwarf, awned spike, waxy leaves, *Rht1* gene
279	BW49929	SUP152/BECARD	CIMMYT, Mexico (CIMMYT line)	CIMMYT, Mexico	Semi-dwarf, awned spike, waxy leaves, *Rht1* gene
280	BW49931	WBLL4/KUKUNA//WBLL1/3/WBLL1*2/BRAMBLING	CIMMYT, Mexico (CIMMYT line)	CIMMYT, Mexico	Semi-dwarf, awned spike, waxy leaves, *Rht1* gene

281	BW49932	ITP40/AKURI	CIMMYT, Mexico (CIMMYT line)	CIMMYT, Mexico	Semi-dwarf, awned spike, waxy leaves, *Rht1* gene
282	BW49933	KIRITATI/WBLL1//MESIA/3/KIRITATI/WBLL1	CIMMYT, Mexico (CIMMYT line)	CIMMYT, Mexico	Semi-dwarf, awned spike, waxy leaves, *Rht1* gene
283	BW49934	KIRITATI/WBLL1//2*BLOUK #1	CIMMYT, Mexico (CIMMYT line)	CIMMYT, Mexico	Semi-dwarf, awned spike, waxy leaves, *Rht1* gene
284	BW49936	SUP152/AKURI//SUP152	CIMMYT, Mexico (CIMMYT line)	CIMMYT, Mexico	Semi-dwarf, awned spike, waxy leaves, *Rht1* gene
285	BW49939	MUTUS*2/AKURI	CIMMYT, Mexico (CIMMYT line)	CIMMYT, Mexico	Semi-dwarf, awned spike, waxy leaves, *Rht1* and *Rht2* genes
286	BW49943	PBW343*2/KUKUNA/3/PASTOR//CHIL/PRL/4/GRACK	CIMMYT, Mexico (CIMMYT line)	CIMMYT, Mexico	Semi-dwarf, awned spike, waxy leaves, *Rht1* gene
287	BW49948	BECARD/FRNCLN	CIMMYT, Mexico (CIMMYT line)	CIMMYT, Mexico	Semi-dwarf, awned spike, waxy leaves, *Rht1* gene
288	BW49949	WBLL1*2/BRAMBLING//CHYAK	CIMMYT, Mexico (CIMMYT line)	CIMMYT, Mexico	Semi-dwarf, awned spike, waxy leaves, *Rht1* gene
289	BW49950	BECARD//ND643/2*WBLL1	CIMMYT, Mexico (CIMMYT line)	CIMMYT, Mexico	Semi-dwarf, awned spike, waxy leaves, *Rht1* gene
290	BW49953	KAUZ*2/MNV//KAUZ/3/MILAN/4/BAV92/5/AKURI/6/MUTUS	CIMMYT, Mexico (CIMMYT line)	CIMMYT, Mexico	Semi-dwarf, awned spike, waxy leaves, *Rht1* gene

291	BW49954	KACHU/BECARD//WBLL1*2/BRAMBLING	CIMMYT, Mexico (CIMMYT line)	CIMMYT, Mexico	Semi-dwarf, awned spike, waxy leaves, *Rht1* gene
292	BW49955	KAUZ/PASTOR//PBW343/3/KIRITATI/4/FRNCLN	CIMMYT, Mexico (CIMMYT line)	CIMMYT, Mexico	Semi-dwarf, awned spike, waxy leaves, *Rht1* gene
293	BW49956	SUP152*2/TECUE #1	CIMMYT, Mexico (CIMMYT line)	CIMMYT, Mexico	Semi-dwarf, awned spike, waxy leaves, *Rht1* gene
294	BW49957	FRANCOLIN #1/AKURI #1//FRNCLN	CIMMYT, Mexico (CIMMYT line)	CIMMYT, Mexico	Semi-dwarf, awned spike, waxy leaves, *Rht1* gene
295	BW49958	ND643/2*TRCH//MUTUS/3/SUP152	CIMMYT, Mexico (CIMMYT line)	CIMMYT, Mexico	Semi-dwarf, awned spike, waxy leaves, *Rht1* gene
296	BW48314	WBLL1*2/4/YACO/PBW65/3/KAUZ*2/TRAP//KAUZ/5/KACHU #1	CIMMYT, Mexico (CIMMYT line)	CIMMYT, Mexico	Semi-dwarf, awned spike, waxy leaves, *Rht1* gene
297	BW49217	ALTAR 84/AE.SQUARROSA (221)//3*BORL95/3/URES/JUN//KAUZ/4/WBLL1/5/REH/HARE//2*BCN/3/CROC_1/AE.SQUARRO	CIMMYT, Mexico (CIMMYT line)	CIMMYT, Mexico	Semi-dwarf, awned spike, waxy leaves, *Rht1* gene
298	BW49307	WBLL1*2/KURUKU*2/5/REH/HARE//2*BCN/3/CROC_1/AE.SQUARROSA (213)//PGO/4/HUITES	CIMMYT, Mexico (CIMMYT line)	CIMMYT, Mexico	Semi-dwarf, awned spike, waxy leaves, *Rht1* gene
299	BW49325	ATTILA*2/PBW65*2/4/BOW/NKT//CBRD/3/CBRD	CIMMYT, Mexico (CIMMYT line)	CIMMYT, Mexico	Semi-dwarf, awned spike, waxy leaves, *Rht1* gene

300	BW49326	ATTILA*2/PBW65*2/4/BOW/NKT//CBRD/3/CBRD	CIMMYT, Mexico (CIMMYT line)	CIMMYT, Mexico	Semi-dwarf, awned spike, waxy leaves, *Rht1* and *Rht2* genes
301	BW49327	ATTILA*2/PBW65*2/4/BOW/NKT//CBRD/3/CBRD	CIMMYT, Mexico (CIMMYT line)	CIMMYT, Mexico	Semi-dwarf, awned spike, waxy leaves, *Rht1* gene
302	BW49328	ATTILA*2/PBW65*2/4/BOW/NKT//CBRD/3/CBRD	CIMMYT, Mexico (CIMMYT line)	CIMMYT, Mexico	Semi-dwarf, awned spike, waxy leaves, *Rht1* gene
303	BW49329	ATTILA*2/PBW65*2/4/BOW/NKT//CBRD/3/CBRD	CIMMYT, Mexico (CIMMYT line)	CIMMYT, Mexico	Semi-dwarf, awned spike, waxy leaves, *Rht1* gene
304	BW49330	ATTILA*2/PBW65*2/4/BOW/NKT//CBRD/3/CBRD	CIMMYT, Mexico (CIMMYT line)	CIMMYT, Mexico	Semi-dwarf, awned spike, waxy leaves, *Rht1* gene
305	BW49331	ATTILA*2/PBW65*2/4/BOW/NKT//CBRD/3/CBRD	CIMMYT, Mexico (CIMMYT line)	CIMMYT, Mexico	Semi-dwarf, awned spike, waxy leaves, *Rht1* gene
306	BW49333	BAV92//IRENA/KAUZ/3/HUITES*2/4/GONDO/TNMU	CIMMYT, Mexico (CIMMYT line)	CIMMYT, Mexico	Semi-dwarf, awned spike, waxy leaves, *Rht1* gene
307	BW49342	KACHU*2//CHIL/CHUM18	CIMMYT, Mexico (CIMMYT line)	CIMMYT, Mexico	Semi-dwarf, awned spike, waxy leaves, *Rht2* gene
308	BW49351	KACHU #1*2/WHEAR	CIMMYT, Mexico (CIMMYT line)	CIMMYT, Mexico	Semi-dwarf, awned spike, waxy leaves, *Rht1* gene
309	BW49385	FRANCOLIN #1//WBLL1*2/BRAMBLING	CIMMYT, Mexico (CIMMYT line)	CIMMYT, Mexico	Semi-dwarf, awned spike, waxy leaves, *Rht1* gene

310	BW49392	BECARD/KACHU	CIMMYT, Mexico (CIMMYT line)	CIMMYT, Mexico	Semi-dwarf, awned spike, waxy leaves, *Rht1* gene
311	BW49394	BECARD/KACHU	CIMMYT, Mexico (CIMMYT line)	CIMMYT, Mexico	Semi-dwarf, awned spike, waxy leaves, *Rht1* gene
312	BW49094	ALTAR 84/AE.SQUARROSA (221)//3*BORL95/3/URES/JUN//KAUZ /4/WBLL1/5/MUTUS	CIMMYT, Mexico (CIMMYT line)	CIMMYT, Mexico	Semi-dwarf, awned spike, waxy leaves, *Rht1* gene
313	BW49400	ALTAR 84/AE.SQUARROSA (221)//3*BORL95/3/URES/JUN//KAUZ /4/WBLL1/5/MUTUS	CIMMYT, Mexico (CIMMYT line)	CIMMYT, Mexico	Semi-dwarf, awned spike, waxy leaves, *Rht1* gene
314	BW49099	KINGBIRD #1//INQALAB 91*2/TUKURU	CIMMYT, Mexico (CIMMYT line)	CIMMYT, Mexico	Semi-dwarf, awned spike, waxy leaves, *Rht1* and *Rht2* genes
315	BW49448	ATTILA*2/PBW65*2//TOBA97/PAST OR	CIMMYT, Mexico (CIMMYT line)	CIMMYT, Mexico	Semi-dwarf, awned spike, waxy leaves, *Rht1* gene
316	BW49456	WBLL1*2/VIVITSI//PRINIA/PASTOR /3/WBLL1*2/BRAMBLING	CIMMYT, Mexico (CIMMYT line)	CIMMYT, Mexico	Semi-dwarf, awned spike, waxy leaves, *Rht1* gene
317	BW49458	NAC/TH.AC//3*PVN/3/MIRLO/BUC/4 /2*PASTOR/5/KACHU/6/KACHU	CIMMYT, Mexico (CIMMYT line)	CIMMYT, Mexico	Semi-dwarf, awned spike, waxy leaves, *Rht1* gene
318	Pasteur	Cadenza//Palermo/KS91WGRC11	Wheat breeding lab, University of Guelph	Netherlands	Hard red spring, awnless spike, waxy leaves, no *Rht* genes
319	Carberry	Alsen/Superb	Wheat breeding lab, University of Guelph	Canada	Red spring, awned spike, *Rht1* gene
320	Norwell	Max/PT742//Bluesky///Max/Coteau//Blu esky	Wheat breeding lab, University of Guelph	Canada	Hard red spring, awned spike, no *Rht* genes
