# Supplementary material for: Population structure of Nepali spring wheat (Triticum aestivum L.) germplasm
Source: BMC Plant Biol. 2020 Nov 23;20:530. doi: 10.1186/s12870-020-02722-8 (PMC7682013; doi:10.1186/s12870-020-02722-8)
Supplement: Supplementary file 2 — Additional file 2 Table S2. Summary of distribution of SNPs in wheat genomes across 21 chromosomes in the Nepali Wheat Diversity Panel. [file 12870_2020_2722_MOESM2_ESM.pdf]

Manuscript title:
Population Structure of Nepali Spring Wheat (*Triticum aestivum* L.) Germplasm

Journal:
BMC Plant Biology

Authors:
*Kamal Khadka[1], Davoud Torkamaneh[1,2,3], Mina Kaviani[1], Francois Belzile[2,3], Manish N. Raizada[1,] and Alireza Navabi[1]

Affiliation:
*[1] Department of Plant Agriculture, University of Guelph, Guelph, Ontario, Canada, N1G 2W1

Corresponding author email address:
kamal.khadka011@gmail.com

Additional file 1: Table S2. Summary of distribution of SNPs in wheat genomes across 21 chromosomes in the Nepali Wheat Diversity Panel

	**Genome**			**Total**
**Chromosomes**	**Genome A**	**Genome B**	**Genome C**	
1	5377	6544	1883	13804
2	6422	8374	2046	16842
3	4881	7806	1247	13934
4	4808	2648	550	8006
5	4494	6647	1013	12154
6	3979	7952	1343	13274
7	7578	8264	1532	17374
Total	37539	48235	9614	95388
