# Supplementary material for: Population structure of Nepali spring wheat (Triticum aestivum L.) germplasm
Source: BMC Plant Biol. 2020 Nov 23;20:530. doi: 10.1186/s12870-020-02722-8 (PMC7682013; doi:10.1186/s12870-020-02722-8)
Supplement: Supplementary file 3 — Additional file 3 Table S3. Assignment of individual accession in the Nepali Wheat Diversity Panel to different subpopulation based on Q-matrix obtained from fastSTRUCTURE. [file 12870_2020_2722_MOESM3_ESM.pdf]

Manuscript title:

Population Structure of Nepali Spring Wheat (Triticum aestivum L.) Germplasm

Journal:

BMC Plant Biology

Authors:

*Kamal Khadka[1], Davoud Torkamaneh[1,2,3], Mina Kaviani[1], Francois Belzile[2,3], Manish N. Raizada[1,] and Alireza Navabi[1]

Affiliation:

*[1] Department of Plant Agriculture, University of Guelph, Guelph, Ontario, Canada, N1G 2W1

Corresponding author email address:

kamal.khadka011@gmail.com

Additional file 3: Table S3. Assignment of individual accession in the Nepali Wheat Diversity Panel to different subpopulation based on Q-matrix obtained from fastSTRUCTURE

Entry	ID	Subpopulation assigned	Q-matrix for K=4			
310	BW49392	2	0.000001	0.999996	0.000001	0.000001
112	NGRC 04410	3	0.06723	0.000001	0.932768	0.000001
311	BW49394	2	0.000001	0.999996	0.000001	0.000001
94	NGRC 02622	3	0.442918	0.000001	0.55708	0.000001
28	NGRC 02472	4	0.000001	0.288455	0.000001	0.711542
128	NGRC 04430	1	0.613955	0.000001	0.386042	0.000001
64	NGRC 02584	4	0.000001	0.000001	0.000001	0.999996
283	BW49934	2	0.045469	0.908875	0.000001	0.045654
191	Pasanglhamu	2	0.000001	0.882494	0.022348	0.095157
167	NGRC 04474	3	0.000001	0.000001	0.999996	0.000001
248	BW49069	2	0.000001	0.999996	0.000001	0.000001
144	NGRC 04450	4	0.000001	0.000001	0.391668	0.608329
202	BW30655	2	0.025076	0.938789	0.036133	0.000001
41	NGRC 02557	3	0.386425	0.000001	0.595937	0.017637
227	BW48140	2	0.000256	0.990452	0.000001	0.00929
99	NGRC 02630	1	0.999996	0.000001	0.000001	0.000001
209	BW45568	2	0.000001	0.999996	0.000001	0.000001
109	NGRC 04406	1	0.999996	0.000001	0.000001	0.000001
182	Annapurna 2	2	0.043254	0.956743	0.000001	0.000001
45	NGRC 02561	4	0.000001	0.000001	0.043952	0.956046
303	BW49329	2	0.000001	0.999996	0.000001	0.000001
51	NGRC 02568	3	0.000001	0.000001	0.999996	0.000001
188	Achyut	2	0.000001	0.939489	0.000001	0.060508
242	BW48162	2	0.000001	0.999996	0.000001	0.000001
273	BW49112	2	0.000001	0.954749	0.000001	0.045248
150	NGRC 04456	2	0.000001	0.999996	0.000001	0.000001
57	NGRC 02575	3	0.134169	0.000001	0.865828	0.000001
18	NGRC 02458	1	0.999996	0.000001	0.000001	0.000001
90	NGRC 02617	1	0.972192	0.000001	0.027805	0.000001

223	BW48136	2	0.000001	0.999996	0.000001	0.000001
186	BL 1135	4	0.017668	0.380052	0.000001	0.602278
9	NGRC 00205	3	0.000001	0.000001	0.999996	0.000001
140	NGRC 04446	3	0.083778	0.000001	0.901894	0.014327
249	BW49227	2	0.000001	0.999996	0.000001	0.000001
29	NGRC 02496	3	0.000001	0.000001	0.999996	0.000001
130	NGRC 04432	3	0.268263	0.000001	0.731734	0.000001
63	NGRC 02582	3	0.000001	0.000001	0.999996	0.000001
298	BW49307	2	0.000001	0.829941	0.001669	0.168388
319	Carberry	4	0.051663	0.367847	0.034703	0.545787
219	BW45595	2	0.000001	0.9108	0.037488	0.051711
260	BW49089	2	0.000001	0.820218	0.000001	0.179779
213	BW45578	2	0.015691	0.917175	0.000001	0.067133
16	NGRC 02456	3	0.346522	0.000001	0.653475	0.000001
284	BW49936	2	0.033709	0.790719	0.019915	0.155656
176	Vinayak	2	0.000001	0.616388	0.000001	0.383609
201	Tilottama	2	0.000001	0.888882	0.000001	0.111115
270	BW49109	2	0.17511	0.673614	0.115546	0.035731
3	NGRC 00179	3	0.06003	0.000001	0.939968	0.000001
208	BW44908	2	0.000001	0.702373	0.020998	0.276627
55	NGRC 02572	3	0.000001	0.000001	0.999996	0.000001
275	BW49922	2	0.005736	0.91925	0.000001	0.075013
281	BW49932	2	0.007153	0.822022	0.000001	0.170823
85	NGRC 02611	1	0.999996	0.000001	0.000001	0.000001
233	BW48150	2	0.012842	0.660921	0.000001	0.326235
308	BW49351	2	0.000001	0.999996	0.000001	0.000001
73	NGRC 02595	4	0.000001	0.120396	0.115633	0.76397
183	Annapurna 3	2	0.038399	0.961598	0.000001	0.000001
160	NGRC 04466	3	0.000001	0.018348	0.540677	0.440974
317	BW49458	2	0.000001	0.999996	0.000001	0.000001
42	NGRC 02558	3	0.383517	0.000001	0.61648	0.000001
157	NGRC 04463	3	0.000001	0.000001	0.999996	0.000001
8	NGRC 00204	3	0.000001	0.000001	0.999996	0.000001
47	NGRC 02564	2	0.000001	0.702277	0.000001	0.29772
212	BW45173	2	0.000001	0.631328	0.07073	0.29794
215	BW45592	2	0.000001	0.999996	0.000001	0.000001
315	BW49448	2	0.000001	0.999996	0.000001	0.000001
14	NGRC 02452	3	0.385022	0.000001	0.614975	0.000001
258	BW49084	2	0.000001	0.999996	0.000001	0.000001
169	Kalyansona	2	0.000001	0.699429	0.000001	0.300569
203	BW35623	2	0.000001	0.660191	0.000001	0.339806
178	Vaskar	2	0.000001	0.873527	0.000001	0.126471
257	BW49344	2	0.000001	0.773478	0.000001	0.22652
141	NGRC 04447	2	0.000001	0.999996	0.000001	0.000001
83	NGRC 02609	4	0.000001	0.000001	0.493544	0.506453
89	NGRC 02615	1	0.999996	0.000001	0.000001	0.000001
113	NGRC 04413	3	0.000001	0.000001	0.999996	0.000001
276	BW49923	2	0.000001	0.999996	0.000001	0.000001
135	NGRC 04439	1	0.484748	0.070068	0.274427	0.170757
56	NGRC 02574	3	0.000001	0.000001	0.999996	0.000001
125	NGRC 04427	2	0.111846	0.529512	0.000001	0.358641
166	NGRC 04473	2	0.000001	0.999996	0.000001	0.000001
116	NGRC 04417	3	0.000001	0.000001	0.999996	0.000001
259	BW49088	3	0.000001	0.000001	0.999996	0.000001
301	BW49327	4	0.035611	0.370952	0.021824	0.571613

153	NGRC 04459	2	0.000001	0.823506	0.000001	0.176491
232	BW48149	1	0.999996	0.000001	0.000001	0.000001
11	NGRC 02449	2	0.000001	0.945863	0.000001	0.054134
300	BW49326	3	0.359342	0.000001	0.640655	0.000001
77	NGRC 02603	2	0.000001	0.924641	0.000001	0.075356
10	NGRC 02448	2	0.036612	0.963385	0.000001	0.000001
145	NGRC 04451	2	0.198288	0.658532	0.000001	0.143178
199	Dhaulagiri	3	0.000001	0.000001	0.999996	0.000001
48	NGRC 02565	2	0.000001	0.999996	0.000001	0.000001
226	BW48171	3	0.000001	0.000001	0.999996	0.000001
306	BW49333	2	0.000001	0.999996	0.000001	0.000001
103	NGRC 04399	2	0.000001	0.999996	0.000001	0.000001
246	BW48169	4	0.000001	0.000001	0.000001	0.999996
62	NGRC 02581	3	0.000001	0.000001	0.999996	0.000001
36	NGRC 02551	2	0.000001	0.999996	0.000001	0.000001
320	Norwell	2	0.000001	0.999996	0.000001	0.000001
218	BW45165	2	0.000001	0.765292	0.000001	0.234705
75	NGRC 02599	4	0.000001	0.181149	0.000001	0.818848
92	NGRC 02620	3	0.000001	0.000001	0.999996	0.000001
272	BW49111	4	0.033021	0.15921	0.077163	0.730606
132	NGRC 04434	3	0.002042	0.000001	0.997955	0.000001
292	BW49955	4	0.000001	0.000001	0.476847	0.52315
184	BL 1022	4	0.000001	0.18599	0.000001	0.814007
221	BW48133	2	0.000001	0.999996	0.000001	0.000001
261	BW49391	1	0.65861	0.000001	0.341388	0.000001
147	NGRC 04453	2	0.003896	0.889542	0.035568	0.070995
224	BW48137	2	0.000001	0.840046	0.000001	0.159952
263	BW49093	2	0.008842	0.849106	0.000001	0.14205
24	NGRC 02466	2	0.000001	0.787369	0.000001	0.212628
60	NGRC 02579	3	0.000001	0.000001	0.999996	0.000001
115	NGRC 04416	2	0.000001	0.758305	0.000001	0.241692
247	BW48168	2	0.000001	0.813883	0.000001	0.186115
299	BW49325	1	0.999996	0.000001	0.000001	0.000001
285	BW49939	4	0.000001	0.000001	0.221658	0.77834
35	NGRC 02550	2	0.000001	0.469712	0.085233	0.445054
174	Lumbini	2	0.000001	0.690828	0.01186	0.297311
133	NGRC 04436	2	0.000001	0.999996	0.000001	0.000001
121	NGRC 04423	1	0.433614	0.210728	0.000001	0.355656
172	NL 30	2	0.000001	0.945479	0.000001	0.054518
253	BW49077	3	0.000001	0.000001	0.999996	0.000001
97	NGRC 02625	3	0.000001	0.000001	0.999996	0.000001
108	NGRC 04405	3	0.000001	0.000001	0.999996	0.000001
277	BW49924	3	0.001835	0.000001	0.998162	0.000001
104	NGRC 04400	3	0.000001	0.000001	0.999996	0.000001
84	NGRC 02610	2	0.000001	0.571072	0.428926	0.000001
316	BW49456	2	0.000001	0.502849	0.087676	0.409473
190	Kanti	2	0.000001	0.999996	0.000001	0.000001
266	BW49097	4	0.000001	0.000001	0.000001	0.999996
54	NGRC 02571	2	0.000001	0.80197	0.000001	0.198027
120	NGRC 04422	3	0.000001	0.000001	0.999996	0.000001
187	Annapurna 4	3	0.271438	0.000001	0.728559	0.000001
280	BW49931	2	0.000001	0.808447	0.000001	0.191551
114	NGRC 04414	2	0.000001	0.630169	0.05236	0.317469
262	BW49092	2	0.050094	0.803962	0.000001	0.145943
168	Lerma 52	4	0.063344	0.248897	0.000001	0.687758

236	BW48155	2	0.000001	0.999996	0.000001	0.000001
111	NGRC 04409	2	0.000001	0.882593	0.000001	0.117404
119	NGRC 04421	2	0.000001	0.999996	0.000001	0.000001
106	NGRC 04402	2	0.000001	0.732176	0.000001	0.267821
15	NGRC 02455	2	0.000001	0.973332	0.000001	0.026665
38	NGRC 02553	3	0.446493	0.000001	0.553504	0.000001
156	NGRC 04462	4	0.000001	0.418709	0.000001	0.581288
200	Danphe	1	0.999996	0.000001	0.000001	0.000001
100	NGRC 02631	3	0.000001	0.000001	0.999996	0.000001
286	BW49943	1	0.999996	0.000001	0.000001	0.000001
123	NGRC 04425	2	0.000001	0.999996	0.000001	0.000001
235	BW48154	1	0.5722	0.085154	0.146293	0.196353
27	NGRC 02471	3	0.000001	0.000001	0.999996	0.000001
129	NGRC 04431	2	0.000001	0.987975	0.000001	0.012022
70	NGRC 02591	3	0.000001	0.000001	0.999996	0.000001
180	NL 251	3	0.000001	0.000001	0.999996	0.000001
105	NGRC 04401	2	0.000001	0.958438	0.022279	0.019282
170	Pitic 62	2	0.000001	0.999996	0.000001	0.000001
239	BW48172	3	0.000001	0.000001	0.999996	0.000001
237	BW48156	2	0.000001	0.999996	0.000001	0.000001
287	BW49948	4	0.000001	0.000001	0.10499	0.895007
252	BW49075	4	0.000001	0.000001	0.000001	0.999996
118	NGRC 04419	3	0.118942	0.119253	0.438735	0.323069
193	Gautam	2	0.000001	0.988109	0.000001	0.011888
229	BW48144	2	0.000001	0.999996	0.000001	0.000001
274	BW49113	4	0.000001	0.194654	0.000001	0.805343
4	NGRC 00180	3	0.000001	0.000001	0.999996	0.000001
127	NGRC 04429	3	0.000001	0.000001	0.999996	0.000001
122	NGRC 04424	2	0.000001	0.999996	0.000001	0.000001
282	BW49933	1	0.999996	0.000001	0.000001	0.000001
264	BW49399	2	0.000001	0.999996	0.000001	0.000001
71	NGRC 02593	3	0.207427	0.000001	0.79257	0.000001
88	NGRC 02614	4	0.223478	0.284567	0.000001	0.491953
164	NGRC 04471	3	0.000001	0.000001	0.999996	0.000001
179	Nepal 297	2	0.000001	0.999996	0.000001	0.000001
53	NGRC 02570	4	0.000001	0.216597	0.000001	0.7834
82	NGRC 02608	3	0.000001	0.000001	0.999996	0.000001
30	NGRC 02544	1	0.492635	0.133866	0.000001	0.373498
206	BW43354	2	0.000001	0.999996	0.000001	0.000001
294	BW49957	3	0.000001	0.000001	0.999996	0.000001
87	NGRC 02613	3	0.000001	0.000001	0.999996	0.000001
107	NGRC 04404	3	0.000001	0.000001	0.999996	0.000001
195	NL 971	2	0.000001	0.999996	0.000001	0.000001
46	NGRC 02563	4	0.000001	0.000001	0.000001	0.999996
13	NGRC 02451	4	0.225456	0.25633	0.050791	0.467422
110	NGRC 04408	3	0.000001	0.000001	0.999996	0.000001
181	Annapurna 1	3	0.000001	0.000001	0.999996	0.000001
288	BW49949	2	0.000001	0.690186	0.000001	0.309811
171	RR21	2	0.000001	0.87341	0.000001	0.126587
138	NGRC 04444	2	0.009773	0.589282	0.000001	0.400944
5	NGRC 00181	2	0.000001	0.970052	0.000001	0.029945
66	NGRC 02586	2	0.000001	0.709486	0.000001	0.290511
312	BW49094	3	0.000001	0.000001	0.999996	0.000001
44	NGRC 02560	2	0.000049	0.980058	0.019892	0.000001
61	NGRC 02580	3	0.229129	0.000001	0.770868	0.000001

23	NGRC 02465	3	0.000001	0.000001	0.999996	0.000001
313	BW49400	2	0.000001	0.59466	0.000007	0.405332
244	BW48166	3	0.000001	0.000001	0.999996	0.000001
12	NGRC 02450	4	0.000001	0.000001	0.000001	0.999996
159	NGRC 04465	3	0.000001	0.000001	0.999996	0.000001
96	NGRC 02624	2	0.000001	0.671682	0.000001	0.328315
134	NGRC 04437	3	0.000001	0.000001	0.999996	0.000001
131	NGRC 04433	2	0.000001	0.669872	0.000001	0.330125
302	BW49328	3	0.000001	0.000001	0.999996	0.000001
175	Tribeni	4	0.000001	0.28188	0.000001	0.718117
295	BW49958	3	0.208129	0.000001	0.791868	0.000001
165	NGRC 04472	3	0.000001	0.000001	0.999996	0.000001
279	BW49929	3	0.21915	0.000001	0.780847	0.000001
251	BW49072	1	0.999996	0.000001	0.000001	0.000001
102	NGRC 02633	2	0.000001	0.662473	0.000001	0.337525
163	NGRC 04470	1	0.551984	0.000001	0.448013	0.000001
21	NGRC 02461	3	0.000001	0.000001	0.999996	0.000001
196	Aditya	2	0.000001	0.999996	0.000001	0.000001
309	BW49385	4	0.000001	0.000001	0.000001	0.999996
139	NGRC 04445	2	0.142692	0.485802	0.046338	0.325168
149	NGRC 04455	4	0.000001	0.225006	0.000001	0.774991
98	NGRC 02629	2	0.000001	0.999996	0.000001	0.000001
39	NGRC 02554	3	0.000001	0.000001	0.999996	0.000001
31	NGRC 02546	4	0.000001	0.22001	0.000001	0.779987
81	NGRC 02607	2	0.033451	0.792673	0.02254	0.151336
124	NGRC 04426	2	0.0229	0.682484	0.000001	0.294614
68	NGRC 02589	3	0.000001	0.000001	0.999996	0.000001
93	NGRC 02621	3	0.000001	0.000001	0.999996	0.000001
256	BW49082	3	0.27337	0.000001	0.726627	0.000001
314	BW49099	3	0.000001	0.000001	0.999996	0.000001
225	BW48139	2	0.055423	0.892314	0.000001	0.052261
69	NGRC 02590	2	0.000001	0.688368	0.000001	0.311629
26	NGRC 02470	3	0.000001	0.000001	0.999996	0.000001
1	NGRC 00176	3	0.000001	0.000001	0.999996	0.000001
268	BW49102	2	0.00936	0.60007	0.000001	0.390568
158	NGRC 04464	3	0.000001	0.000001	0.999996	0.000001
117	NGRC 04418	2	0.020656	0.52073	0.458613	0.000001
290	BW49953	2	0.000001	0.999996	0.000001	0.000001
2	NGRC 00177	4	0.000001	0.000001	0.000001	0.999996
189	Rohini	2	0.000001	0.999996	0.000001	0.000001
91	NGRC 02619	2	0.000001	0.994948	0.000001	0.005049
207	BW44829	1	0.572075	0.095318	0.000001	0.332605
86	NGRC 02612	4	0.046027	0.000001	0.230811	0.723161
136	NGRC 04440	2	0.051273	0.521426	0.000001	0.4273
72	NGRC 02594	4	0.000001	0.000001	0.000001	0.999996
43	NGRC 02559	2	0.000001	0.468547	0.091384	0.440068
7	NGRC 00202	1	0.999996	0.000001	0.000001	0.000001
197	Vijay	2	0.000001	0.795105	0.002602	0.202292
243	BW48163	3	0.031856	0.000001	0.968141	0.000001
148	NGRC 04454	3	0.081633	0.000001	0.918364	0.000001
19	NGRC 02459	2	0.000001	0.999996	0.000001	0.000001
152	NGRC 04458	2	0.000001	0.999996	0.000001	0.000001
58	NGRC 02576	3	0.025145	0.000001	0.974852	0.000001
173	UP 262	1	0.999996	0.000001	0.000001	0.000001
151	NGRC 04457	4	0.030541	0.412789	0.000572	0.556098

204	BW43945	2	0.000325	0.980523	0.019151	0.000001
177	Siddhartha	2	0.000001	0.999895	0.000001	0.000102
126	NGRC 04428	2	0.000001	0.999996	0.000001	0.000001
80	NGRC 02606	2	0.013695	0.714944	0.000001	0.27136
293	BW49956	3	0.000001	0.000001	0.999996	0.000001
65	NGRC 02585	3	0.000001	0.000001	0.999996	0.000001
25	NGRC 02467	2	0.000001	0.845388	0.0189	0.13571
296	BW48314	2	0.000001	0.959926	0.000001	0.040071
22	NGRC 02462	2	0.000001	0.905829	0.000001	0.094168
238	BW48158	2	0.000001	0.999996	0.000001	0.000001
245	BW48165	2	0.000001	0.68325	0.000001	0.316747
143	NGRC 04449	2	0.000001	0.918791	0.000001	0.081207
240	BW48174	2	0.008883	0.896411	0.000001	0.094705
192	BL 1473	4	0.000001	0.000001	0.000001	0.999996
198	Gaura	4	0.000001	0.000001	0.000001	0.999996
305	BW49331	3	0.000001	0.000001	0.999996	0.000001
155	NGRC 04461	3	0.000001	0.000001	0.999996	0.000001
222	BW48135	3	0.04327	0.099204	0.69136	0.166166
185	Bhrikuti	2	0.000748	0.989812	0.000001	0.009438
40	NGRC 02556	2	0.000001	0.918793	0.000001	0.081204
137	NGRC 04443	2	0.000001	0.88111	0.003491	0.115398
76	NGRC 02602	4	0.000001	0.000001	0.017692	0.982305
142	NGRC 04448	2	0.000001	0.999996	0.000001	0.000001
146	NGRC 04452	3	0.430681	0.000001	0.569317	0.000001
228	BW48141	2	0.000334	0.754422	0.245243	0.000001
161	NGRC 04467	3	0.000001	0.000001	0.543876	0.456121
33	NGRC 02548	3	0.000001	0.000001	0.999996	0.000001
32	NGRC 02547	2	0.000001	0.975167	0.000001	0.024831
211	BW45573	3	0.000001	0.000001	0.999996	0.000001
265	BW49095	2	0.000001	0.989296	0.000001	0.010701
255	BW49079	3	0.081509	0.030062	0.843805	0.044624
101	NGRC 02632	4	0.038345	0.351566	0.000001	0.610087
234	BW48151	2	0.000001	0.999996	0.000001	0.000001
217	BW45587	3	0.000001	0.000001	0.999996	0.000001
297	BW49217	4	0.000001	0.000001	0.000001	0.999996
307	BW49342	2	0.000001	0.859869	0.000001	0.140128
59	NGRC 02578	3	0.000001	0.000001	0.999996	0.000001
220	BW48132	4	0.000001	0.184845	0.000001	0.815153
79	NGRC 02605	3	0.000001	0.000001	0.999996	0.000001
6	NGRC 00199	2	0.000001	0.999996	0.000001	0.000001
250	BW49235	2	0.000001	0.999996	0.000001	0.000001
95	NGRC 02623	3	0.392987	0.000001	0.60701	0.000001
216	BW45593	3	0.000001	0.000001	0.999996	0.000001
50	NGRC 02567	2	0.000001	0.999996	0.000001	0.000001
49	NGRC 02566	2	0.000001	0.707248	0.000001	0.292749
254	BW49078	3	0.000001	0.000001	0.999996	0.000001
241	BW48161	2	0.000001	0.999996	0.000001	0.000001
52	NGRC 02569	4	0.000001	0.000001	0.104724	0.895273
210	BW45152	2	0.000001	0.977019	0.000001	0.022979
194	WK1204	2	0.000001	0.999996	0.000001	0.000001
205	BW45161	3	0.048471	0.145181	0.496952	0.309396
289	BW49950	2	0.008288	0.92509	0.024079	0.042543
278	BW49927	4	0.02998	0.401004	0.000001	0.569015
230	BW48145	2	0.023046	0.798355	0.000001	0.178598
154	NGRC 04460	2	0.000001	0.999996	0.000001	0.000001

37	NGRC 02552	3	0.000001	0.000001	0.999996	0.000001
304	BW49330	2	0.000001	0.999996	0.000001	0.000001
291	BW49954	2	0.000001	0.778016	0.000001	0.221981
271	BW49110	2	0.02132	0.948721	0.029958	0.000001
67	NGRC 02587	3	0.000001	0.000001	0.999996	0.000001
74	NGRC 02596	3	0.000001	0.000001	0.846913	0.153084
269	BW49108	2	0.000001	0.817022	0.000001	0.182975
214	BW45590	3	0.000001	0.000001	0.999996	0.000001
17	NGRC 02457	2	0.000001	0.900959	0.090108	0.008932
318	Pasteur	4	0.000001	0.000001	0.000001	0.999996
78	NGRC 02604	3	0.000001	0.000001	0.999996	0.000001
34	NGRC 02549	3	0.000001	0.000001	0.999996	0.000001
20	NGRC 02460	2	0.000001	0.838974	0.000001	0.161023
231	BW48148	2	0.000001	0.836216	0.000001	0.163782
