# Supplementary material for: Population structure of Nepali spring wheat (Triticum aestivum L.) germplasm
Source: BMC Plant Biol. 2020 Nov 23;20:530. doi: 10.1186/s12870-020-02722-8 (PMC7682013; doi:10.1186/s12870-020-02722-8)
Supplement: Supplementary file 4 — Additional file 4 Table S4. The frequency of genotypes in the Nepali Wheat Diversity Panel as differentiated into different subpopulations based on Q-matrix obtained from fastSTRUCTURE. [file 12870_2020_2722_MOESM4_ESM.pdf]

Manuscript title:
Population Structure of Nepali Spring Wheat (*Triticum aestivum* L.) Germplasm

Journal:
BMC Plant Biology

Authors:
*Kamal Khadka[1], Davoud Torkamaneh[1,2,3], Mina Kaviani[1], Francois Belzile[2,3], Manish N. Raizada[1,] and Alireza Navabi[1]

Affiliation:
*[1] Department of Plant Agriculture, University of Guelph, Guelph, Ontario, Canada, N1G 2W1

Corresponding author email address:
kamal.khadka011@gmail.com

Additional file 4: Table S4. The frequency of genotypes in the Nepali Wheat Diversity Panel as differentiated into different subpopulations based on Q-matrix obtained from fastSTRUCTURE

	**Subpopulation 1**	**Subpopulation 2**	**Subpopulation 3**	**Subpopulation 4**	**Total**
**Landraces**	12 (0.07)	62 (0.37)	70 (0.42)	22 (0.13)	166 (1.00)
**CIMMYT lines**	8 (0.07)	69 (0.60)	25 (0.22)	13 (0.11)	115 (1.00)
**Released varieties**	2 (0.06)	22 (0.65)	4 (0.12)	6(0.18)	34 (1.00)
**Canadian varieties**	0	1 (0.33)	0	2 (0.67)	3 (1.00)

Note: values in parentheses indicate the frequency within each seed source category
